# Our national nutrient reduction needs: Applying a conservation prioritization framework to US agricultural lands

**DOI:** 10.1016/j.jenvman.2023.119758

**Published:** 2023-12-12

**Authors:** Lily Kirk, Jana E. Compton, Anne Neale, Robert D. Sabo, Jay Christensen

**Affiliations:** a Oak Ridge Institute for Science and Education – US Environmental Protection Agency (EPA), 109 T.W. Alexander Drive, Durham, NC, 27709, USA; b US EPA, Office of Research and Development, Center for Public Health and Environmental Assessment, Pacific Ecological Systems Division, Corvallis, OR, 97330, USA; c US EPA, Office of Research and Development, Center for Public Health and Environmental Assessment, Public Health and Environmental Systems Division, Durham, NC, USA; d US EPA, Office of Research and Development, Center for Public Health and Environmental Assessment, Health and Environmental Effects Division, Washington, DC, USA; e US EPA, Office of Research and Development, Center for Environmental Measurement and Modeling, Watershed and Ecosystem Characterization Division, Cincinnati, OH, USA

**Keywords:** Targeting, Nutrient surplus, Nutrient use efficiency, In-field management, Edge-of-field buffer, Wetland restoration

## Abstract

Targeted conservation approaches seek to focus resources on areas where they can deliver the greatest benefits and are recognized as key to reducing nonpoint source nutrients from agricultural landscapes into sensitive receiving waters. Moreover, there is growing recognition of the importance and complementarity of in-field and edge-of-field conservation for reaching nutrient reduction goals. Here we provide a generic prioritization that can help with spatial targeting and applied it across the conterminous US (CONUS). The prioritization begins with identifying areas with high agricultural nutrient surplus, i.e., where the most nitrogen (N) and/or phosphorus (P) inputs are left on the landscape after crop harvest. Subwatersheds with high surplus included 52% and 50% of CONUS subwatersheds for N and P, respectively, and were located predominantly in the Midwest for N, in the South for P, and in California for both N and P. Then we identified the most suitable conservation strategies using a hierarchy of metrics including nutrient use efficiency (proportion of new nutrient inputs removed by crop harvest), tile drainage, existing buffers for agricultural run-off, and wetland restoration potential. In-field nutrient input reduction emerged as a priority because nutrient use efficiency fell below a high but achievable goal of 0.7 (30% of nutrients applied are not utilized) in 45% and 44% of CONUS subwatersheds for N and P, respectively. In many parts of the southern and western US, in-field conservation (i.e., reducing inputs + preventing nutrients from leaving fields) alone was likely the optimal strategy as agriculture was already well-buffered. However, stacking in-field conservation with additional edge-of-field buffering would be important to conservation strategies in 35% and 29% of CONUS subwatersheds for N and P, respectively. Nutrient use efficiencies were often high enough in the Midwest that proposed strategies focused more on preventing nutrients from leaving fields, managing tile effluent, and buffering agricultural fields. Almost all major river basins would benefit from a variety of nutrient reduction conservation strategies, underscoring the potential of targeted approaches to help limit excess nutrients in surface and ground waters.

## 1. Introduction

The degradation of aquatic ecosystems due to excess nitrogen (N) and phosphorus (P) has been documented repeatedly and globally (e.g., Conley et al., 2009) and is a prime example of a “wicked” environmental problem (Patterson et al., 2013). Excess nutrients released to the environment originate mostly from nonpoint agricultural sources (Brown and Froemke, 2012; Carpenter et al., 1998) and vary across the conterminous United States (CONUS) (Dubrovsky et al., 2010; Hong et al., 2013). The amelioration of excess nutrients is generally addressed by voluntary adoption of conservation practices by individual farmers and landowners supported in large part by USDA (United States Department of Agriculture) as well as US EPA (Environmental Protection Agency) and state programs. With limited resources, targeted conservation approaches that focus resources on areas with the greatest potential impact are widely seen as necessary to achieve local to national water quality goals regarding nutrient reduction (Fleming et al., 2022; Conservation Effects Assessment Project, 2022; Ribaudo, 1989; Shortle et al., 2012). Targeted approaches have most often been applied to smaller subwatersheds (Tomer et al., 2013; Zimmerman et al., 2019), but modeling suggests effectiveness of spatial targeting at coarser scales for water quality improvements in terms of both nutrient reduction (Cheng et al., 2020; Dagnew et al., 2019; Evenson et al., 2021) and cost (Perez et al., 2014; Rabotyagov et al., 2014). To that end, federal conservation programs have incorporated spatial targeting in their program designs, for example through USDA-NRCS (Natural Resources Conservation Service) Landscape Conservation Initiatives that dedicate funds from conservation programs to projects in priority watersheds (Perez and Walker, 2014) or through EPA’s Clean Water Act § 319 Nonpoint Source Management Program where states, territories, and tribes can prioritize watersheds through their respective programs (USEPA, 2013). Currently there is a large effort to fund agricultural conservation practices through the Bipartisan Infrastructure Law (White House, 2022) and Inflation Reduction Act (Fischer and Outlaw, 2022); fine-tuning prioritizations to help direct these funds is critical to maximizing the nutrient reduction benefit of this large infusion of federal dollars.

The factors that inform the selection of priority areas for nutrient pollution amelioration vary by program and state, so describing agricultural conservation needs at the US national scale remains a challenge, but useful for both context and adaptive management. Priority watershed selection factors include, but are not limited to, river nutrient loads, impaired waters status, source water protection, institutional readiness, and likelihood of improvement (USEPA, 2013). Whereas degraded watersheds are usually prioritized, sometimes watersheds are selected to protect pristine natural areas. These differences in selection factors and local priorities mean a national map of conservation needs could not easily be stitched together from state prioritizations; instead, an analysis using consistent metrics is needed. To our knowledge, no spatially resolved national prioritization of nutrient reduction conservation in agriculture has been published. In addition, states and federal programs periodically revisit their watershed prioritizations, especially as environmental conditions change (e.g., climate change) and the data informing them improves. As states and programs strategize on how to reduce the most nutrients, systematic national conservation maps could be used to confirm or improve current prioritizations.

Effective prioritization of conservation to address nonpoint source nutrient pollution begins with identification of the areas that need additional measures to combat excess nutrients. Nutrient source areas that generate disproportionately high N and/or P loads in water bodies are often determined using watershed-scale hydrologic models (White et al., 2014) or from direct measurements of stream nutrient loads (if the data are available and adequate). An alternative – yet complementary – approach relies on indirect measurements that may signal high nutrient loads (Fleming et al., 2022): For agricultural landscapes, agricultural nutrient surplus (i.e., estimates of the balance of nutrients left by agricultural operations; henceforth “surplus”) (MacDonald et al., 2011; McLellan et al., 2018; Quemada et al., 2020), may serve that function well (Dupas et al., 2015), especially at CONUS scales where intensive modeling or stream monitoring networks are less available. The emergence of spatially detailed national nutrient inventories that calculate surplus based on empirical estimates of nutrient inputs and outputs (Swaney and Howarth, 2019; Swaney et al., 2018) can help inform spatial targeting efforts for nutrient pollution amelioration at coarse spatial scales. Modeling efforts have shown that more nutrient reduction would likely occur nationally if high surplus areas were prioritized for conservation implementation than if surplus was not considered (e.g., Cheng et al., 2020).

A myriad of conservation practices are available to help reduce nutrient loads from agricultural operations, so in addition to high nutrient input areas, information about the most suitable type of conservation to combat excess nutrients in any given area is also very useful to the prioritization process. Water quality is driven by three key processes along a pollutant transfer continuum – source, mobilization, and delivery – along which nonpoint source pollution may be reduced (Granger et al., 2010; Lintern et al., 2018). NRCS promotes a systems approach to agricultural nutrient reduction that is based on a similar conceptual framework: It begins with avoiding nutrient excesses, then controlling their transport, and finally trapping nutrients at the water’s edge (Osmond et al., 2019). This systems approach incorporates two key ideas: First, in-field conservation (i.e., managing nutrients in the field) is closer in space and time to nutrient inputs than edge-of-field conservation (i.e., intercepting agricultural run-off and filtering the nutrients out before they reach surface waters), and thus may be better at reducing nutrient excesses. This in-field then edge-of-field hierarchy is similarly promoted by the Agricultural Conservation Planning Framework (ACPF), which is gaining traction for prioritizing conservation at local scales in the Midwest and beyond. ACPF promotes “in field” practices before “below field” ones, with different considerations when the dominant water flowpath is tile drainage vs surface run-off (Tomer et al., 2013). A conceptual framework similar to NRCS’s systems approach and ACPF would be appropriate at coarse scales, although recommendations for general types of conservation (vs individual practices) would be most realistic for regional and national prioritization efforts.

The second key idea of NRCS’s systems approach is “stacking” or combining conservation practices to maximize nutrient removal (Dagnew et al., 2019; Law et al., 2022; Rabotyagov et al., 2014; Tomer, 2018). In-field and edge-of-field practices are broadly stackable with each other (Christianson et al., 2018). Stacked conservation bolsters the inefficiencies of each type of conservation. For example, buffers can be surprisingly leaky with nutrients (Flanagan and Richardson, 2010), highlighting the importance of reducing in-field surplus alongside edge-of-field conservation. Conversely, there is growing recognition that in-field conservation may not be enough to mitigate the current and legacy effects of agriculture on water nutrient concentrations (Delgado, 2016), and additional edge-of-field conservation may be necessary to achieve nutrient reduction goals (Rabotyagov et al., 2014). Stacking conservation whenever possible allows the practices to work synergistically in space and time (Roley et al., 2016) and may be the best use of conservation dollars (Christianson et al., 2018) if practices are judiciously recommended based on regional/local conditions (Macrae et al., 2021).

The goal of this study was to prioritize subwatersheds across CONUS for nutrient reduction conservation, and thus provide a synoptic assessment (broad perspective instead of detailed analysis) (McAllister et al., 2000) of nutrient reduction conservation needs for the nation. In this study, we leveraged recently available agricultural nutrient metrics and other CONUS-wide agricultural landscape data, applied a prioritization framework consistent with others and adapted for coarse scales, and identified both priority areas for N and P reduction as well as the conservation strategies best suited to tackle the nutrient problems within subwatersheds. We identified subwatersheds where conservation efforts can focus on reducing nutrient inputs, managing drainage effluent, or creating edge-of-field buffers, as well as subwatersheds where these can be stacked to have the greatest impact toward reducing nutrient release into surface waters. While our spatial analysis illustrates a generalized national example using relatively large subwatersheds, the framework is adjustable for many spatial scales, and the customizable dataset can be filtered for a region of interest. Together, the framework and dataset can be an additional tool in a watershed manager’s toolbox.

## 2. Methods

### 2.1. Prioritization approach

**Fig. 1.**
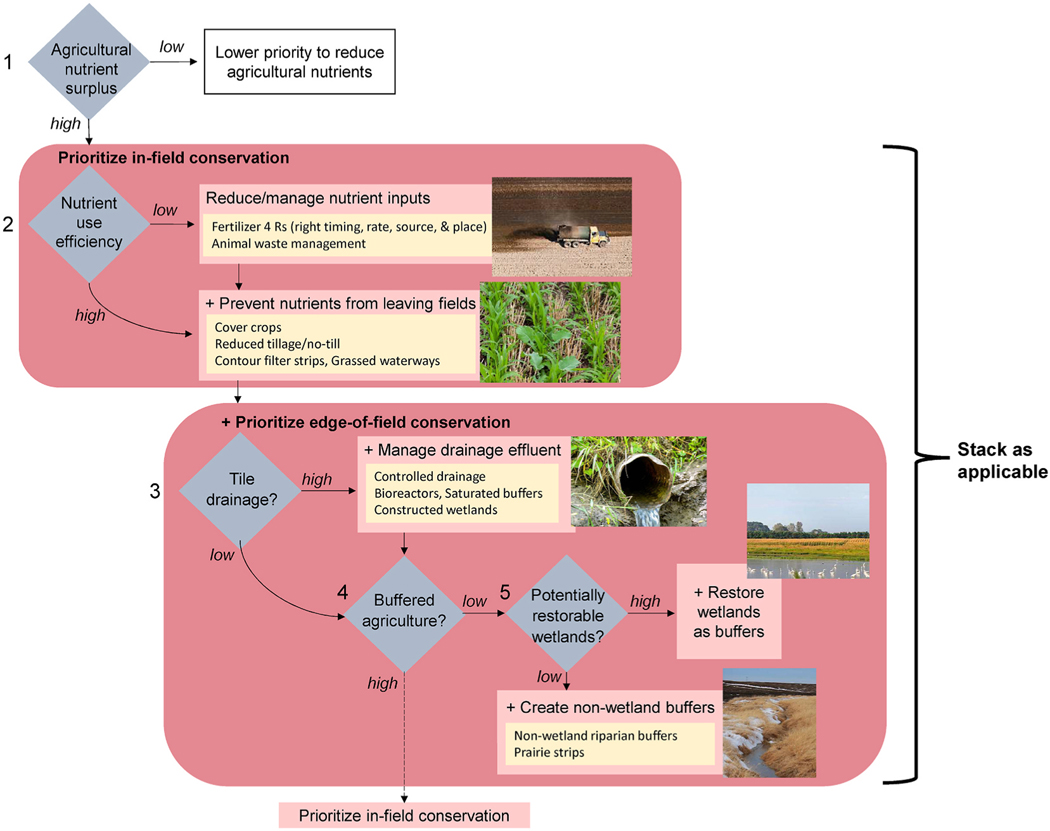
Prioritization approach for nutrient conservation strategies.

Our approach to prioritize nutrient reduction conservation is illustrated in Fig. 1. The starting point for prioritization was identifying areas that were likely to generate high nutrient loads using the agricultural nutrient surplus metric. Surplus is an empirical estimate of how much nutrients are left on the landscape after crop harvest (see Table 1 and Supplementary Materials T1 for details on its calculation). Only high surplus subwatersheds were selected for subsequent analysis (Fig. 1, step 1, choice of thresholds used discussed in 2.3).

The remaining steps determine the most appropriate conservation strategy and borrow conceptually from NRCS’s systems approach and ACPF. Using nutrient use efficiencies, we were able to differentiate where it would be most beneficial for conservation to focus on reducing/managing nutrient inputs (e.g., changes to fertilizer rates or timing) (Fig. 1, step 2). Nutrient use efficiency (henceforth, “efficiency”) is the proportion of the new nutrient inputs that is incorporated into crop harvest and livestock. Low efficiencies indicate opportunities for reducing nutrient inputs in the field by better management of fertilizer and animal waste (often termed “nutrient management”). Because most of the surplus that is responsible for degrading water quality comes from unutilized or excess fertilizer and manure inputs, it is logical to maximize in-field efficiency even when implementing other types of conservation. In areas where efficiencies are high yet surplus remains high due to pervasive and intensive agriculture, more attention and resources might be allocated to prevent excess nutrient loss from the fields (e.g., via conservation tillage or cover crops) and edge-of-field conservation.

For those areas where edge-of-field conservation that traps or treats excess nutrients in run-off seemed advantageous, we then considered the amount of tile drainage within agricultural fields to identify subwatersheds where conservation practices aimed at intercepting tile flow (e.g., saturated buffers, constructed wetlands) would be instrumental for nutrient reduction (Fig. 1, step 3). Edge-of-field practices could have a larger impact on nutrient inputs into surface waters where agriculture lacks adequate buffers; thus, those areas were prioritized (Fig. 1, step 4). Finally, because of the demonstrated cost-effectiveness of wetland restoration at reducing nutrient loads (see Supplementary Materials T4), we identified subwatersheds that had many wetland restoration opportunities based on soils, slope, and hydrology (i.e., potentially restorable wetlands, or PRW; Fig. 1, step 5).

Because the sources (e.g., synthetic fertilizer vs manure) and transport (e.g., dissolved or sediment-bound) of excess N and P may differ from one another (Sharpley et al., 2000) – and by extension, their mitigation – we chose to carry out the prioritizations for N and P separately (Sharpley et al., 2006). However, managers must be cautious about possible pollution swapping between nutrients when recommending conservation (Sharpley et al., 2006), so we present both results together to better inform decision-makers.

### 2.2. Input datasets & data processing

We used data summarized by 8-digit hydrologic unit code, or HUC8, for our analysis. HUC8s (also known as “subwatersheds” in this paper) are part of a nested hierarchical drainage basin classification system based on surface hydrological features. The system, created by the US Geological Survey, divides the US into progressively smaller drainage units (Jones et al., 2022). In this study, we refer to HUCs at three levels: HUC2s (N = 18), HUC8s (N ~ 2100), and HUC12s (N ~ 83,000).

We primarily used data from the National Nutrient Inventory (NNI) (Sabo et al., 2019, 2021b) and EnviroAtlas (Pickard et al., 2015) (both developed by EPA researchers) (Table 1) in our prioritization effort. We limited our analysis to the 2092 HUC8 subwatersheds (USEPA, 2018) that had data for all metrics (essentially excluded coastal subwatersheds with <1.5% land area). For tile drainage, we aggregated the 30 × 30 m resolution AgTile-US raster (Valayamkunnath et al., 2020) to a 150 × 150 m raster to speed up processing without sacrificing too much resolution (resolution likely sufficient for the HUC8 scale used in our analysis). This lower resolution AgTile-US layer was then overlain with the EnviroAtlas HUC8 layer, and the Intersection area was extracted for each subwatershed. We calculated percent tile drainage by dividing the metric by agricultural area and multiplying by 100. We did ensure that data layers that were combined to create metrics used in the analysis (e. g., PRW, % Cropland, and % Pasture was combined to create PRW as a percentage of agricultural land) had temporal scopes that matched.

**Fig. 2.**
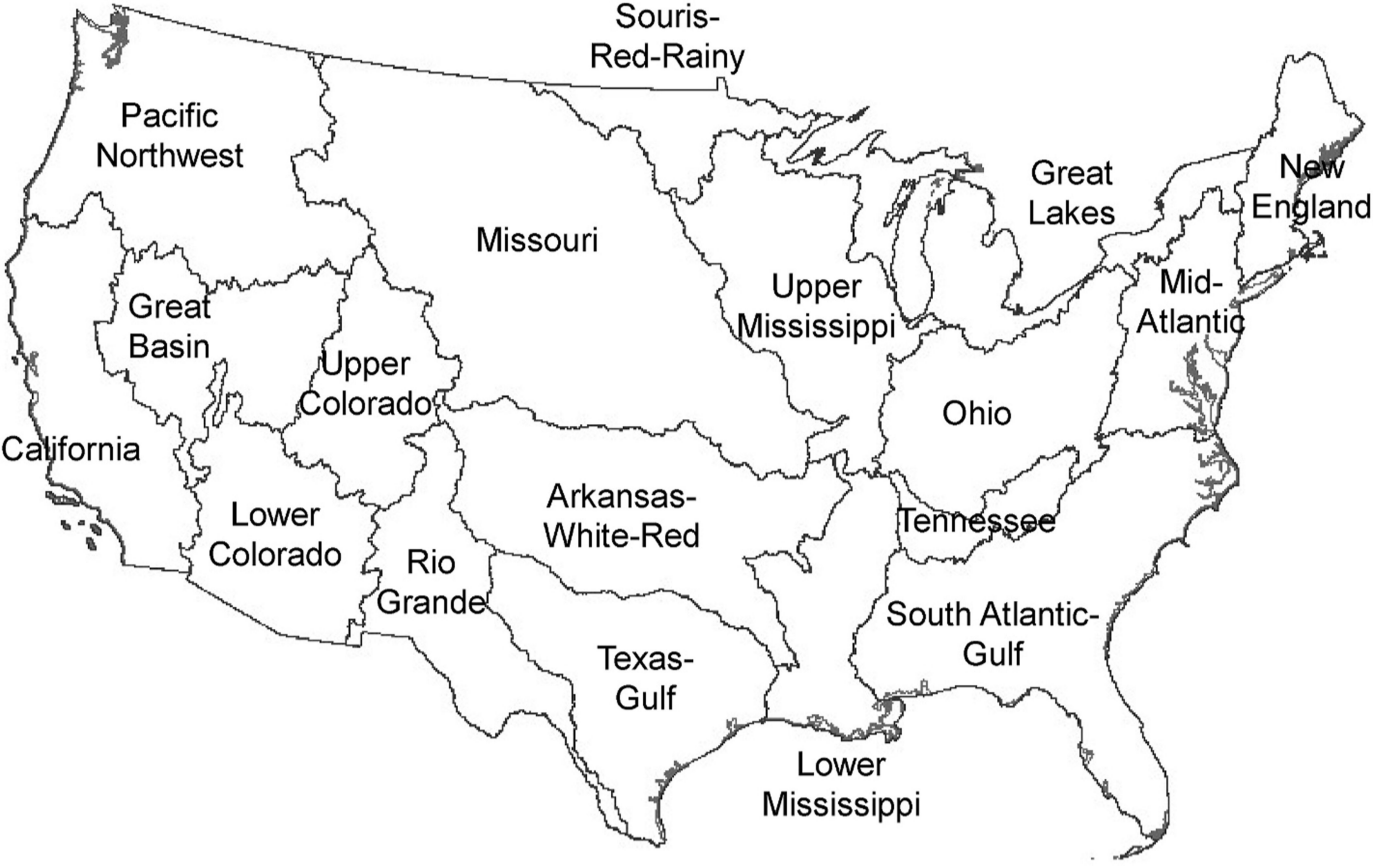
HUC2 river basins of the conterminous US, used to group the HUC8 data presented later.

To help describe metrics and their overlap, a Spearman rank correlation matrix was created for the metrics (measured in area, e.g., km^2^ of tile drainage vs. % tile drainage) for the CONUS data at the HUC8 scale (Table S2), and medians and interquartile ranges (IQR) for metrics (measured in %) were summarized (Fig. S6) by HUC2 river basin (also known as "basin" in this paper) (Fig. 2). Additionally, subwatershed counts for each step of the prioritization (Fig. 1) were tallied. All geospatial and statistical analyses were conducted in R (version 4.1.3) with relevant packages.

### 2.3. Thresholds used for prioritization

In this study, we applied the prioritization approach outlined in Fig. 1 to HUC8 subwatersheds in CONUS, choosing thresholds appropriate for a national analysis. However, because choice of thresholds can greatly influence the prioritization results, the dataset provided allows customization of thresholds in similar analyses based on the scale needed. Greater context to the chosen thresholds and breakpoints for the color classes used in figures is provided in the Supplementary Materials T2.

#### 2.3.1. Agricultural nutrient surplus

We considered N surplus ≥7 kg-N ha^− 1^ y^− 1^ and P surplus ≥1 kg-P ha^− 1^ y^− 1^ as “high” surplus for our analysis. Both reflect median surplus in CONUS, as well as the high end of atmospheric deposition in this dataset, which suggests where surplus was caused by local land management (vs ambient environmental inputs). Only high surplus subwatersheds were prioritized for nutrient reduction in our analysis. Whereas fluxes per unit cropland area are often reported in the literature especially from field-scale studies, the NNI data was reported as fluxes per HUC8 watershed area to better reflect the local landscape and likely impacts on watershed loads (Swaney et al., 2018).

#### 2.3.2. Nutrient use efficiency

We used an efficiency threshold of 0.7 which reflects the ~75th percentiles across CONUS for both nitrogen use efficiency (NUE) and phosphorus use efficiency (PUE) in the NNI data, as well as the global NUE average promoted by Zhang et al. (2015) to meet environmental stewardship goals without compromising food security. The 70% efficiency threshold used in this exercise is not necessarily an end goal for efficiency across the United States (see Supplementary Materials T3), but a reasonable and achievable goal that most subwatersheds should be able to attain under current crop and livestock production practices. Below 70%, farmers can improve efficiencies by reducing nutrient inputs (in-field conservation) that also reduces surplus. At or above 70%, farmers are already operating efficiently in the fields, yet if surplus remains high due to prevalent and intensive agriculture, employing both in-field (especially preventing nutrients from leaving fields) and edge-of-field conservation would be logical.

#### 2.3.3. Tile drainage

We considered subwatersheds where ≥30% of the agricultural area was tiled (roughly the 95th percentile for CONUS) as “highly” tiled areas where conservation is likely to focus on managing drainage effluent.

#### 2.3.4. Nonbuffered agriculture

We considered the subwatersheds with <2% nonbuffered agriculture (40th percentile in CONUS) to have adequate buffers where the focus remains on in-field conservation (and drainage effluent management, as necessary). Subwatersheds with ≥2% nonbuffered agriculture were opportunities for edge-of-field buffering. To provide context to these values, the median percentage of agricultural land in a subwatershed is only 16% (IQR: 3–41%). We chose to use percentage of watershed area (versus percentage of agricultural land) because the same area of unbuffered agriculture in a subwatershed with very little agriculture probably affects the total downstream export of nutrients much less than in an agriculture-heavy subwatershed.

We use the term “buffer” in a broad sense to refer to any natural vegetation along run-off flowpaths between agricultural operations and surface water bodies. Buffers do not have to be immediately adjacent to streams (although that is common) and are not referring to specific NRCS conservation practices. This metric indicates whether agricultural run-off is buffered, not whether water bodies have buffers around them.

#### 2.3.5. Potentially restorable wetlands (PRW)

We considered subwatersheds where PRW were ≥10% of agricultural land to be places where conservation strategies could emphasize wetland restoration. 10% PRW roughly reflects the median PRW for subwatersheds in CONUS. Because we used the PRW metric that was exclusive to agricultural lands (PctPRWAg in EnviroAtlas), we assume PRW have proximity to and can treat the run-off from agricultural lands. Subwatersheds where PRW were not as prevalent, yet edge-of-field conservation would be beneficial, may be areas to focus on other types of conservation such as non-wetland buffers.

## 3. Results

**Fig. 3.**
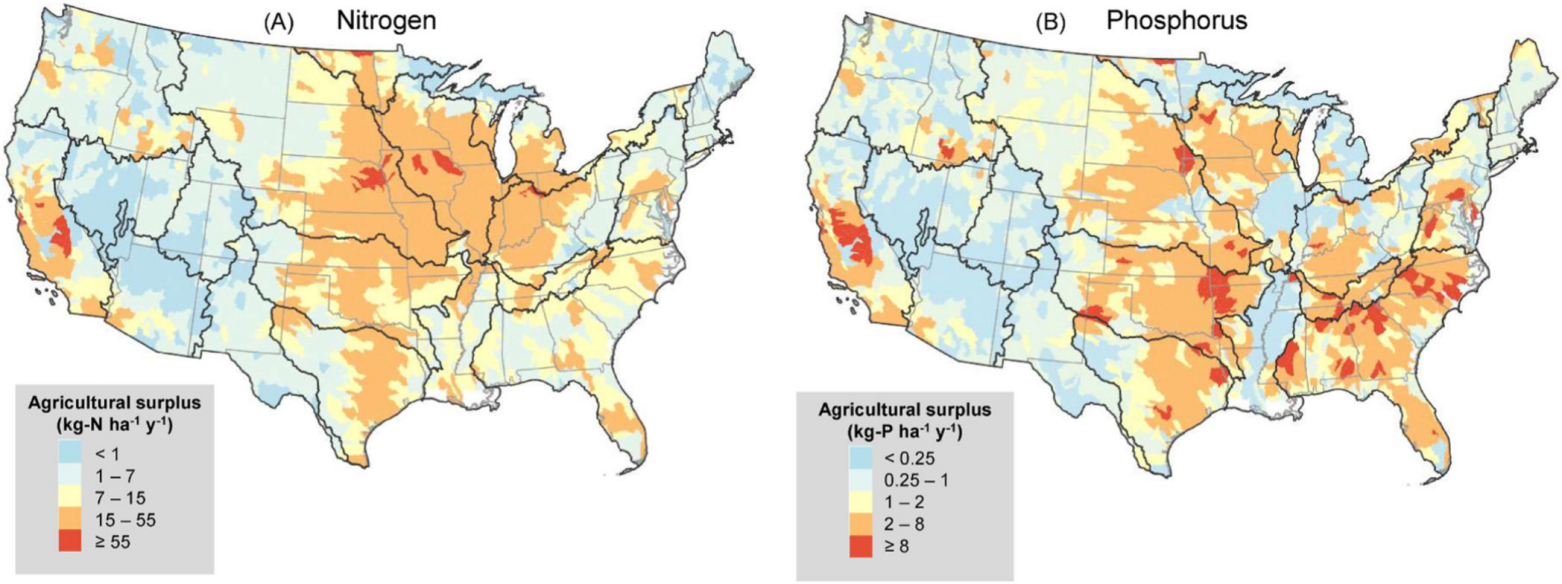
Agricultural nutrient surplus for nitrogen (A) and phosphorus (B) across the conterminous US given as an average yearly surplus per unit area in each HUC8 subwatershed. Based on a chosen threshold for surplus, the user can decide whether to proceed to the next step of the prioritization (Fig. 1, step 1). For this study, we considered surpluses ≥7 kg-N ha^− 1^ y^− 1^ or ≥1 kg-P ha^− 1^ y^− 1^ as “high surplus” and were prioritized for nutrient reduction conservation. States are outlined in light grey and HUC2 river basins (Fig. 2) are outlined in black in this and subsequent maps.

Surplus varied greatly among the 2092 HUC8 subwatersheds in CONUS (Fig. 3 and Fig. S3). As expected, N surplus was correlated to agricultural land area (r = 0.68) across subwatersheds. However, P surplus was only correlated to pasture area (r = 0.36) versus agricultural land in general (see Fig. S1 for spatial distribution of types of agriculture). N and P surpluses were often co-located (r = 0.65), but P surpluses were low (and sometimes negative, indicating soil mining) in the agriculture-heavy Lower Mississippi, Upper Mississippi, and Ohio HUC2 river basins that simultaneously had high N surpluses. The lowest two nutrient surplus classes for N and P (Fig. 3) prevalent in the arid West and New England were left out of further prioritization since the need to improve water quality resulting from agricultural nutrients was likely low at this scale compared to the rest of the country. High N surpluses above 7 kg-N ha^− 1^ y^− 1^ were present in 51.7% of CONUS subwatersheds, and high P surpluses above 1 kg-P ha^− 1^ y^− 1^ were present in 50.0%; these were included in subsequent analyses. The highest surpluses nationwide were in the Midwest predominantly for N, in the South for P, and in California for both N and P. These high surplus subwatersheds represented 89.1% and 97.6% of the net CONUS N and P surpluses, respectively.

**Fig. 4.**
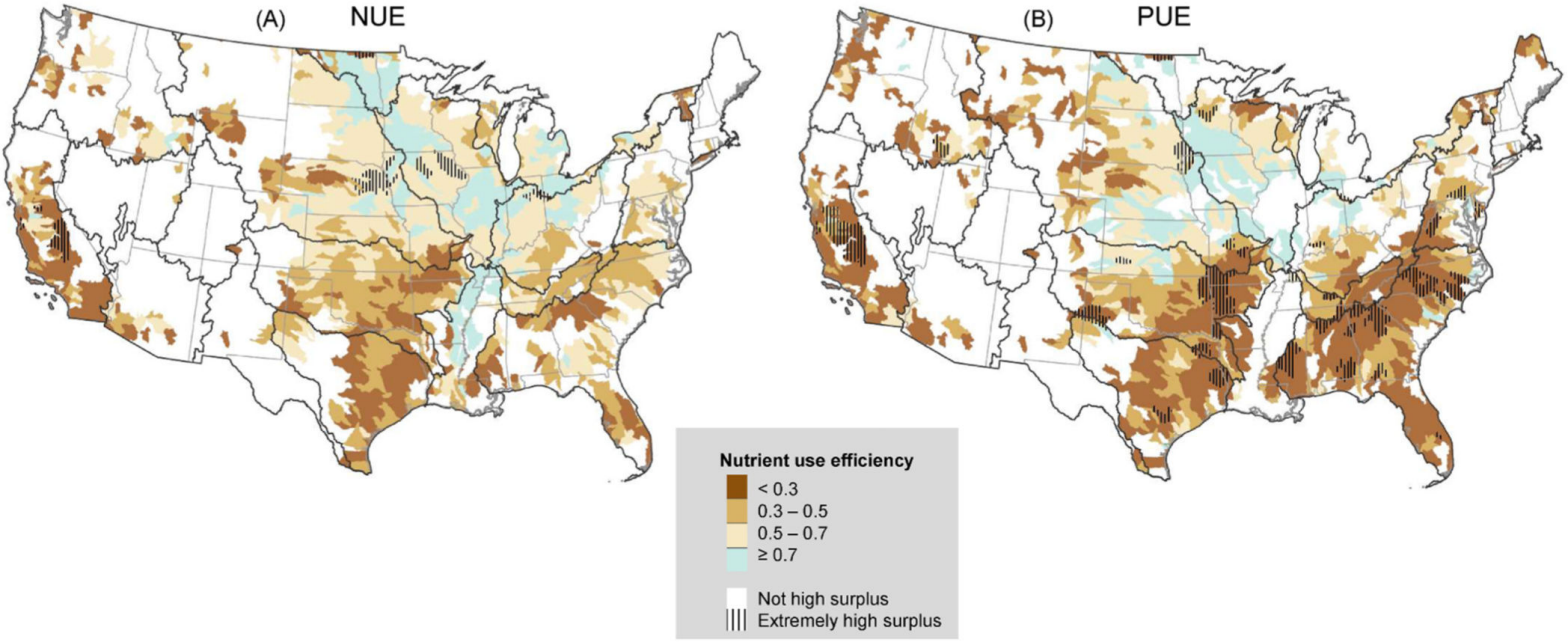
Nitrogen use efficiency (NUE) and phosphorus use efficiency (PUE) for high surplus HUC8 subwatersheds. Subwatersheds with extremely high surpluses (≥55 kg-N ha^− 1^ y^− 1^ or ≥8 kg-P ha^− 1^ y^− 1^) are hatched. We used an efficiency of 0.7 to differentiate between subwatersheds that could benefit from conservation strategies focused on in-field nutrient input reduction (<0.7) versus edge-of-field conservation (≥0.7) (Fig. 1, step 2).

Overall, efficiencies were generally low but also varied across CONUS. The median NUE and PUE for CONUS subwatersheds was 0.46 (IQR: 0.26–0.65) and 0.41 (IQR: 0.19–0.72), respectively. NUE and PUE were well-correlated to each other nationally (r = 0.91). PUE had a negative relationship to P surplus (r = − 0.36), but NUE had little relation to N surplus (r = 0.10). Of the high surplus subwatersheds, 85.8% and 87.8% had efficiency below 0.7, for N or P, respectively (Fig. 4). Of the high N surplus subwatersheds, 49.3% had NUE below 0.5, whereas an even higher 68.3% of high P surplus subwatersheds had PUE below 0.5. These lowest efficiency subwatersheds were located primarily in the South and California and included some of the highest surplus subwatersheds in the country, especially for P (Fig. 4).

**Fig. 5.**
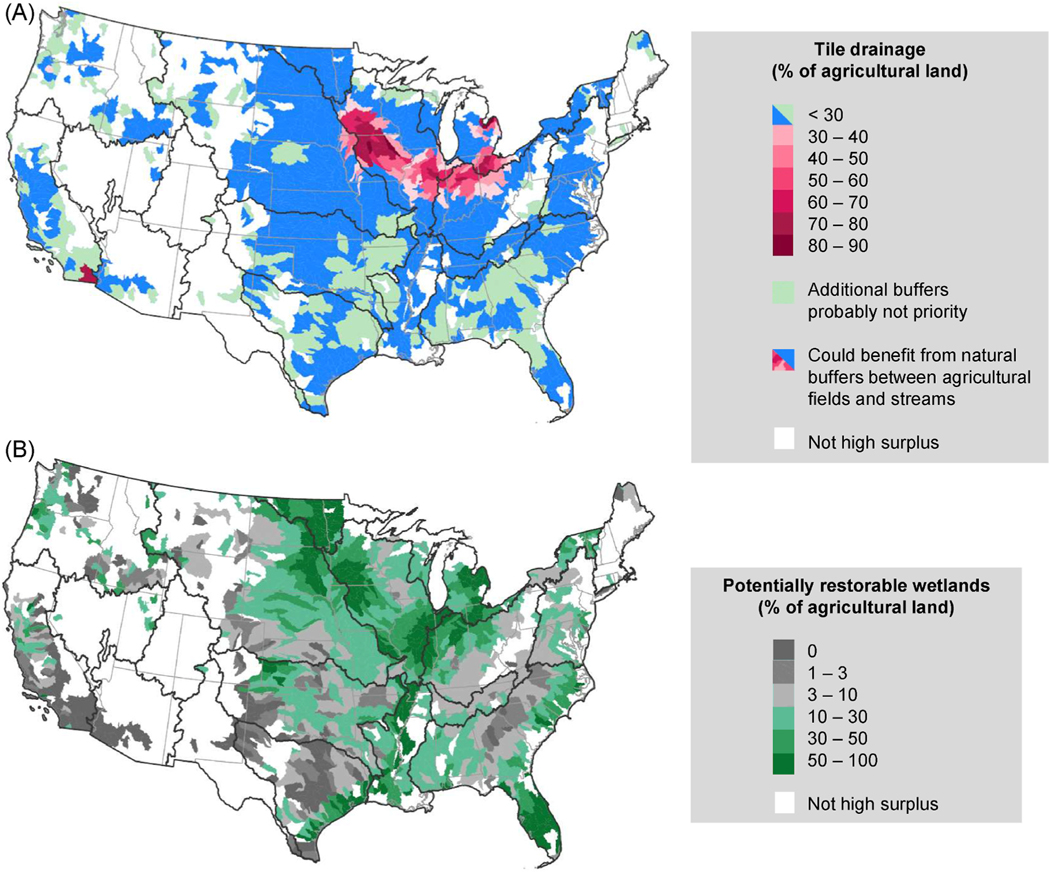
A) Water flowpath concerns for prioritization of edge-of-field conservation in high surplus HUC8 subwatersheds. Managing drainage effluent is important in pink subwatersheds because ≥30% of agricultural lands are drained by tile (Fig. 1, step 3). In light green subwatersheds, nonbuffered agriculture only makes up a small proportion (<2%) of the subwatershed, so conservation efforts can focus on in-field conservation (vs edge-of-field buffering in blue and pink; Fig. 1, step 4). B) More potentially restorable wetlands on agricultural lands (PRW ≥10%, dark green) indicate areas where conservation strategies can focus on restoring wetlands (Fig. 1, step 5). (For interpretation of the references to color in this figure legend, the reader is referred to the Web version of this article.)

The 223,000 km^2^ of tile drainage across CONUS was correlated to agricultural area (r = 0.73) and N surplus (r = 0.62) and less so to P surplus (r = 0.25) across subwatersheds. Nearly all the tile drainage area (98.2%) fell in high N and/or P surplus subwatersheds. Tile drainage was by far most prevalent in the Midwest (Fig. 5A and Fig. S4), but other high surplus subwatersheds where tile drainage was ≥30% of agricultural land included the southern tip of California and northwest Oregon. In total, 115 high surplus subwatersheds were highly tiled.

Like tile drainage, the vast majority (86.8%) of the 741,000 km^2^ of CONUS nonbuffered agricultural lands was in high surplus subwatersheds. Nonbuffered agriculture was highly correlated to agricultural area (r = 0.91), N surplus (r = 0.62), and tile drainage area (r = 0.72) across subwatersheds. The Midwest had the most nonbuffered agriculture (Fig. S5), and these lands also tended to be tile-drained (Fig. S4). Agriculture in many places of the South, Maine, and the West was well-buffered. Of the 314 high surplus subwatersheds with <2% nonbuffered agriculture, all agriculture was buffered in 177 subwatersheds. Three-quarters of the 1266 high N and/or P surplus subwatersheds had ≥2% nonbuffered agriculture, and 837 of them had <30% tile drainage of their agricultural lands (Fig. 5A).

Across CONUS, 514,000 km^2^ or 27.1% of agricultural lands are suitable for wetland restoration, with 89.5% of these lands found in high surplus subwatersheds (Fig. 5B). PRW area was highly correlated to agricultural area (r = 0.80), tile drainage area (r = 0.69), nonbuffered agriculture area (r = 0.79), and N surplus (r = 0.55) across subwatersheds. PRW are most common in the Midwest, along the lower Mississippi River, and in Florida (Fig. 5B). Of high surplus subwatersheds, 58.5% have greater than one-tenth of agricultural lands as PRW. Little to no PRW are found in the Southwest, along with many subwatersheds in the West, central and western Texas, southern Appalachians, and Maine. In the Midwest, many subwatersheds with high PRW also have high tile-drainage (Fig. 5). The Souris-Red-Rainy river basin and Florida offer the greatest opportunities for restoring wetlands to buffer agricultural run-off, in terms of high surplus, PRW, and lack of existing buffers.

**Fig. 6.**
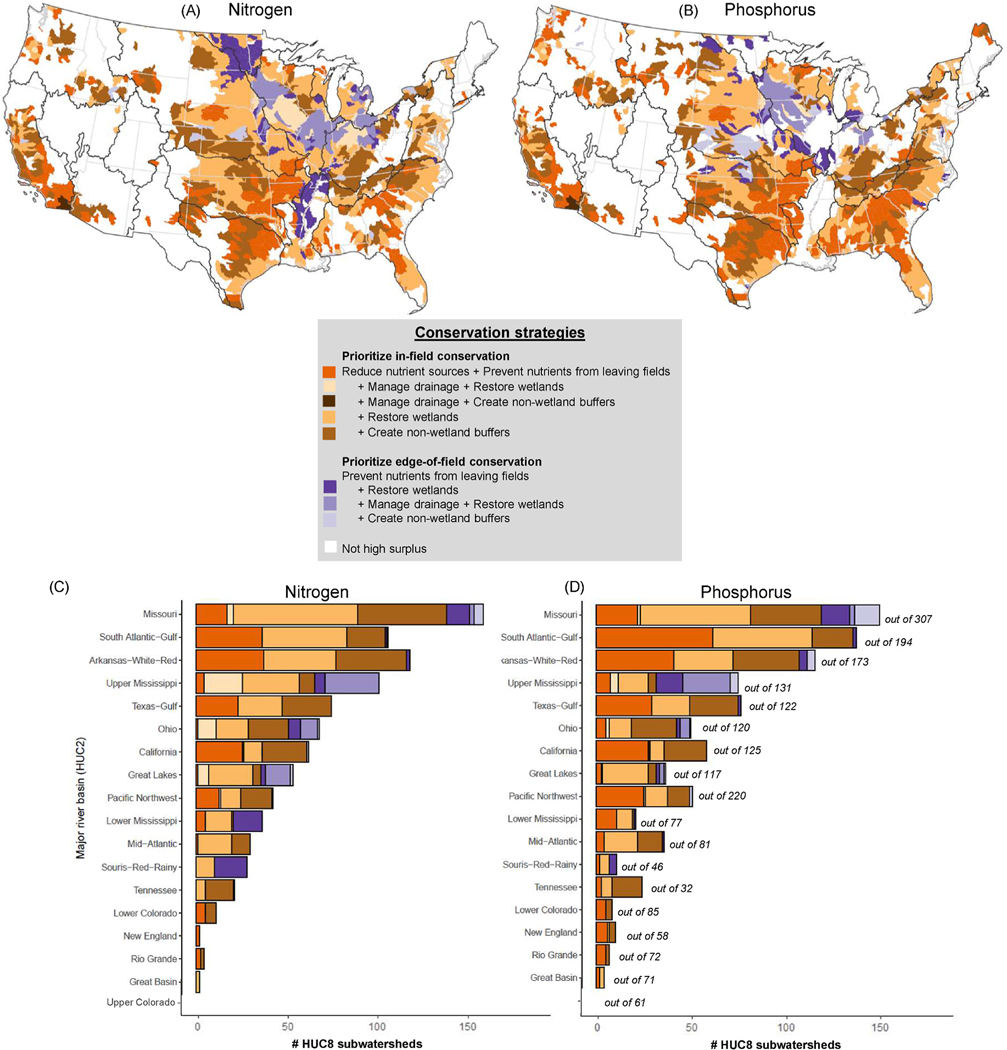
Opportunities to reduce nitrogen (A) and phosphorus (B) inputs to surface waters by agricultural conservation strategy in high surplus HUC8 subwatersheds. All colors represent subwatersheds with high N and/or P surplus. Following the flowchart in Fig. 1, conservation strategy prioritization began with in-field conservation before considering edge-of-field conservation. Subwatersheds were tallied by HUC2 river basin for the different conservation strategies for nitrogen (C) and phosphorus (D). The total number of subwatersheds in each basin is also included in D. (For interpretation of the references to color in this figure legend, the reader is referred to the Web version of this article.)

Combining these metrics following the flowchart in Fig. 1, we were able to delineate not only where there was likely a high need for reducing nutrient inputs into surface waters, but also strategize about what types of stacked conservation might realize nutrient load reductions most effectively. Due to low (<0.7) efficiencies, in-field conservation dominated strategies across high surplus subwatersheds (orange/brown colors in Fig. 6). In contrast, conservation strategies focused first on edge-of-field conservation was generally projected to be best in <15% of high surplus subwatersheds (purples in Fig. 6). A breakdown of subwatersheds in each basin falling under each of these conservation strategies is tallied in Fig. 6C and D. In the following paragraphs, we tally CONUS subwatersheds by conservation strategy:

Strategies focused on in-field nutrient management could reduce excess N for 928 (85.8%) of the 1082 high N surplus subwatersheds. In 724 of those 928 subwatersheds, stacking in-field with edge-of-field conservation was the best strategy to ameliorate high N surplus. Stacking wetland restoration with in-field conservation made sense in 432 of the 724 subwatersheds (light orange/cream in Fig. 6A), with 49 of the 432 also necessitating treatment of drainage effluent as part of the conservation strategy (cream color). In 292 of the 724 subwatersheds, stacking non-wetland buffers with in-field conservation is an appropriate conservation strategy (brown). One subwatershed in southern California (dark brown in Fig. 6A and B) was too arid for wetlands, but in-field conservation stacked with management of drainage effluent and non-wetland buffers was the optimal strategy to stop extremely high N and P surpluses from reaching surface waters. Edge-of-field conservation was not a priority for 22.0% or 204 of the 928 subwatersheds as they were already well-buffered and lacked extensive tile drainage (orange). On the other hand, edge-of-field conservation stacked with conservation practices that kept N from leaving fields was the dominant strategy for nutrient reduction in 14.2% or 154 of the 1082 high N surplus subwatersheds. Conservation strategies could focus on restoring wetlands as buffers in 142 of the 154 subwatersheds (dark/medium purple), located in the Missouri, Lower Mississippi, and Souris-Red-Rainy basins, and 65 of the 142 also likely required managing drainage effluent (medium purple). Twelve other subwatersheds scattered across CONUS would benefit from conservation strategies focused on creating non-wetland buffers in addition to preventing N from leaving fields (light purple in Fig. 6A).

Similar proportions emerged among the strategies for P reduction: Conservation strategies focused on in-field P input reduction were ideal in 87.9% or 919 out of 1046 high P surplus subwatersheds (orange/brown colors in Fig. 6B), and the optimal strategy was to stack in-field and edge-of-field conservation in 66.4% of the 919 subwatersheds. This included strategies that stacked wetland restoration with in-field conservation in 339 subwatersheds (light orange/cream), and additionally stacking with drainage effluent management in 11 (cream color). Non-wetland buffers could be stacked with in-field P management as part of conservation strategies in 271 subwatersheds (brown). Preduction efforts with a focus only on in-field conservation occurred in 309 subwatersheds (orange), mostly due to low PUE but also including one subwatershed in Texas (HUC8: 12050006, also orange) with high PUE but little unbuffered agriculture. Edge-of-field conservation stacked with measures that prevent P from leaving fields was integral to nutrient reduction in 12.2% or 128 of the 1046 high P surplus subwatersheds. Three-quarters of them (97 of the 128) were suitable for conservation strategies that included restoring wetlands as buffers and were located mainly in the Upper Mississippi basin (dark/medium purple). Of the 97, conservation strategies included managing drainage effluent in 42 subwatersheds. Non-wetland buffers were more appropriate as part of P strategies where the focus was mainly on edge-of-field conservation in 30 subwatersheds, mostly in the Missouri, Upper Mississippi, and Arkansas-White-Red basins (light purple).

## 4. Discussion

Recent modeling efforts show that spatial targeting of agricultural conservation practices at large watershed scales can optimize both nutrient load reduction to receiving waters (Dagnew et al., 2019), as well as the cost-effectiveness of practice implementation (Rabotyagov et al., 2014). These authors’ optimization scenarios demonstrate the utility of stacking conservation practices to realize nutrient reduction goals. Our efforts delineated not only which subwatersheds nationwide could be prioritized to reduce nutrients based on agricultural nutrient surplus (52% and 50% of CONUS HUC8 subwatersheds for N and P, respectively), but also what conservation strategies (i.e., combinations of different types of conservation practices) are likely to be effective in each subwatershed. Edge-of-field conservation (such as buffers, wetland restoration, or management of drainage effluent) is often secondary to in-field conservation (reducing/managing nutrient sources + preventing nutrients from leaving fields) when trying to achieve lasting, sustainable nutrient reduction in agricultural landscapes; hence, we utilized nutrient use efficiency as a metric to roughly differentiate between these differing conservation needs. Our analysis underscores the many opportunities (45% and 44% of CONUS subwatersheds for N and P, respectively) to improve in-field nutrient input reduction nationwide. In roughly 1 out of 3 CONUS subwatersheds (35% and 29% for N and P, respectively), stacking in-field with edge-of-field conservation is a strategy with potential to maximize nutrient reduction.

### 4.1. Using nutrient surplus for spatial prioritization

To our knowledge, this study is the first to systematically incorporate surplus and efficiency into prioritizations of nutrient reduction efforts for US agricultural areas, allowing us to advance prioritization work towards water quality goals. Conceptually, surplus is a measure of the potential for nutrients to be delivered to water bodies (and thus, environmental pressure), not a measure of actual nutrient delivery, although the two should converge over long time periods (Oborn et al., 2003). Surplus and nutrient delivery have been found to be strongly correlated (de Wit and Behrendt, 1999; Dupas et al., 2015; Stackpoole et al., 2021), although other factors may affect the relationship (Oenema et al., 2005; Stackpoole et al., 2021). Some nutrients may not arrive at surface waters because they are retained on agricultural land or along the water flowpath to downstream water bodies, so whereas surplus may reflect improvements in in-field nutrient management, it largely misses the effects of other implemented conservation practices (e.g., Stackpoole et al., 2021). Surplus may also differ from nutrient loads delivered to streams because some nutrients percolate into groundwater reservoirs (e.g., aquifers). Random forest models have found N surplus to be a strong predictor of drinking water nitrate violations across the US (Pennino et al., 2020), supporting the use of the surplus metric for prioritization work regarding groundwater, in addition to surface waters. Surplus is relatively simple to calculate based on empirical estimates (e.g., recorded fertilizer sales) versus modeled nutrient loads (with their attendant complexities, inaccuracies, and uncertainties) that are more commonly used in the US. The incorporation of surplus into conservation programs has precedent: Surplus (or nutrient “balance”) has been used widely in the European Union as a performance indicator for agriculture and as a policy instrument (Oenema et al., 2005; Powell et al., 2010). In this study, we chose to use surplus because it was a publicly available dataset for CONUS, but for regions where other data is available, the authors recommend surplus as a complementary metric to nutrient loads.

### 4.2. Nutrient use efficiencies underscore the primacy of in-field conservation

In-field conservation, particularly reducing nutrient inputs, emerged as the most common and essential component of conservation strategies in the subwatersheds needing nutrient reduction due to pervasive low (<0.7) efficiencies across CONUS. Even in the high surplus subwatersheds that have met the efficiency goal for one nutrient, 55.1% of them could improve efficiency in the other nutrient. Based on the same underlying data as surplus, efficiency nevertheless shifts the focus from amounts of excess nutrients to how efficiently agricultural operations utilize new nutrients. The concept of nutrient use efficiency has been used extensively in agronomy from plant (e.g., McDonald et al., 2013) to cropping system scales (e.g., Zhang et al., 2015), and has potential in the conservation realm (Powell et al., 2010). Although the efficiency metric does not identify which processes in the agricultural system cause the inefficiencies, we found that it was useful to help focus conservation strategies based on reducing nutrients at their source (in-field) and ameliorating excess nutrients after they leave the field (edge-of-field).

There is broad agreement that reducing surplus and increasing efficiency through in-field nutrient management is critical for addressing the dual challenges of food security and environmental degradation globally (Schroder et al., 2003¨; Schroder et al., 2011; Zhang et al., 2015). In-field conservation can address the underlying cause of excess nutrients with nutrient management via the 4 Rs (applying the right fertilizer type, at the right rate, at the right time, and in the right place) (Roberts, 2007). As a preventative measure, the 4 Rs have some environmental benefits in addition to reducing nutrient loads to surface waters like reducing legacy nutrient build-up in soils and the potential flux to groundwater. In-field nutrient management may be the most cost-effective way to address excess nutrients (Hansen et al., 2012): Practices like applying less fertilizer often save the farmer money but should be supported with good assessments of current nutrient applications. Successful efforts to decrease in-field N and P surpluses have been achieved for areas draining to the Chesapeake Bay, and monitoring/modeling efforts suggests nutrient loads to the Bay have declined in response (Sabo et al., 2022; Zhang et al., 2022). Beyond nutrient management, in-field conservation attempts to prevent nutrients from leaving the field through practices like cover crops and reduced/no tillage before edge-of-field conservation is even necessary, and these practices are universally applicable regardless of efficiency. From a social perspective, promoting in-field conservation involves farmers in ameliorating nonpoint-source pollution instead of disconnecting farmers from pollution mitigation.

Our finding of the primacy of in-field conservation in achieving nutrient reduction agrees with other prioritizations based on USDA’s Conservation Effects Assessment Project (CEAP) data. In their Mississippi river basin optimization simulations, Rabotyagov et al. (2014) found edge-of-field conservation practices alone could not meet water quality goals without spatial targeting, and even with targeting, the stacked in-field/edge-of-field strategy was key to their cost optimization scenarios. More recently, a CEAP report (Conservation Effects Assessment Project, 2022) indicated that subsurface N and soluble P losses to the environment increased between the first (2003–2006) and second (2013–2016) CEAP surveys, while nutrient management on cropland declined. Informed by data like these, NRCS has prioritized fertilizer and manure management, and our analysis provides a spatial approach that could help with this prioritization.

In this study, we sorted subwatersheds into the in-field vs edge-of-field-focused strategy based on whether they met a high, but feasible efficiency threshold of 0.7. Zhang et al. (2015) actually suggest the US should have NUE goal set slightly above other regions of the world at 0.75, but local/regional conditions and available practices are more likely to dictate efficiency goals (Swaney et al., 2018; Zhang et al., 2015). With the provided dataset, the user can adjust efficiency thresholds if they have differing needs than what is afforded in this analysis. The nuances and consequences of selecting efficiency thresholds are discussed in the Supplementary Materials T3. Some areas may have high surplus alongside high efficiencies even though that is counter-intuitive: In these areas, there are such large amounts of nutrient inputs (usually an intensively farmed area) that any inefficiencies in nutrient use – however slight – result in substantial amounts of nutrients left over. Thus, it is recommended that surplus and efficiency be used together as measures of sustainability (Gerber et al., 2014). As agricultural production-oriented metrics (Johnston and Bruulsema, 2014), surplus and efficiency are easily understood by farmers and particularly suited for discussing in-field conservation such as nutrient management.

### 4.3. Water drainage flowpath shapes edge-of-field conservation strategies

Strategies that emphasize edge-of-field conservation need to consider surface and subsurface flowpaths to increase the likelihood of higher nutrient removal rates. Many subwatersheds in the Midwest are heavily tile-drained, and conservation strategies should lean heavily on managing and treating drainage effluent, for example using saturated buffers, constructed wetlands, denitrifying bioreactors, two-stage ditches, or controlled drainage (Carstensen et al., 2020). This may be in addition to edge-of-field buffering, depending on the percentage of agricultural land that is tiled (Fig. 5A, pink areas). It is also critical to implement in-field conservation in tiled areas (i.e., stacking), especially because intense precipitation events often overwhelm the capacity of drainage management systems (e.g., Kroger et al., 2007), sending effluent directly into bodies of water. In this study, we focused on tile drainage, but other agricultural drainage system such as ditches were not included in the Ag-Tile dataset, and may be more common in certain regions such as the Atlantic Coastal Plain (Needelman et al., 2007). However, the same logic that we used for tile drainage would apply to other artificial drainage systems as well.

If agricultural run-off follows natural flowpaths, conservation strategies can prioritize edge-of-field buffering in areas where current natural vegetation along the flowpath between agricultural fields and water bodies are scarce (and conversely, in well-buffered landscapes, conservation can focus instead on reducing nutrient inputs and preventing nutrients from leaving fields in the first place). Vegetation in buffers slows down water, allowing sediments – and the nutrients bound to them – to settle out. At watershed scales, buffers remove about half of N leaving agricultural fields in the Southeast (Christensen et al., 2013) and less for P (Dagnew et al., 2019; Hanief and Laursen, 2019), and their effectiveness obviously hinges on whether lateral flow draining agricultural fields are routed through the buffer (Wigington et al., 2005) as dispersed sheet flow (Pankau et al., 2012) on their way to bodies of water, as well as residence time of water in the buffer (Cheng et al., 2020). We selected subwatersheds with higher proportions of nonbuffered agriculture (≥2% of subwatershed) for focus on edge-of-field buffering in the conservation strategies, but the coarser input data we used for our national analysis could only identify the presence of buffers ≥30 m. Although 30 m with favorable slope conditions is the recommendation from a meta-analysis by Zhang et al. (2010), such a wide buffer may be less realistic in intense agricultural areas (Hickey and Doran, 2004). A meta-analysis found that the mere presence of riparian buffers seems to positively affect water quality (Mayer et al., 2007), so finer scale data could identify the presence of narrower buffers and thereby downgrade the importance of edge-of-field conservation. However, additional buffers may still be desirable in areas where agricultural fields are already buffered (light green in Fig. 5A) if surpluses are very high but buffer widths are small, given evidence that wide buffers (*>*50 m) are more efficient zones for N removal than narrow buffers (0–25 m; Mayer et al., 2007). There is evidence that landscape position may an even more important consideration than buffer width: Johnson et al. (2013) found buffers adjacent to streams (i.e., riparian buffers) are more effective than their upslope counterparts at reducing nutrients. Future work may be able to develop and include a metric delineating riparian buffer potential.

### 4.4. Targeting and stacking are key to wetland restoration efforts that aim to reduce nutrients

Whereas restored wetlands have the potential to prevent a large amount of nutrients from reaching surface waters (see Supplementary Materials T4), targeted placement of these wetlands is necessary to increase the likelihood of higher nutrient removal rates (Cheng et al., 2020). The general alignment of excess nutrients (i.e., surplus) with landscape suitability for wetlands (i.e., PRW) across CONUS subwatersheds reflects strong correlations of each to agricultural intensity: *>*17% of current US cropland was historically drained (Blann et al., 2009). Correlations are strongest in the Midwestern states, and potential removal there is supported by various modeling efforts both when tile is not considered (Cheng et al., 2020) and when it is considered (Evenson et al., 2021). In watersheds outside of the Midwest, targeting wetland restoration efforts becomes critical because the need for nutrient removal is not as well-aligned with PRW.

Our analysis shows that many of the subwatersheds with high PRW can also benefit greatly from improvements to nutrient use efficiency; thus, stacked in-field/edge-of-field conservation would be the best strategy in these subwatersheds. Wetland restoration is one of the most highly stackable conservation practices (Christianson et al., 2018) and can certainly complement in-field conservation efforts such as nutrient management. Importantly, restored wetlands work best under baseflow conditions when there is higher water retention time, but there is evidence most nutrient transport may occur during high flow (Kamrath and Yuan, 2023; Law et al., 2022), especially as climate change alters the intensity, frequency, and duration of precipitation (Trenberth et al., 2003). Hence, reducing nutrient inputs in places where wetlands are being restored may be as important as wetland restoration itself. Moreover, the potential release of legacy P must be a consideration when restoring wetlands (see Supplementary Materials T4 and Fig. S7) and is another reminder of the primacy of reducing in-field nutrient inputs before relying on edge-of-field conservation.

### 4.5. Broad-scale spatial targeting is a starting point

With the emergence of continental-scale datasets, we are better able to describe conservation needs for the nation and identify the watersheds and strategies to solve large-scale water quality problems (see Supplementary Materials T5 for other considerations). An objective, systematic analysis of national nutrient reduction needs may be useful in a few ways: It can inform federal agencies and legislators as they allocate funds for conservation in the agricultural sector. States and regional entities can also use such a conservation map to confirm their own watershed prioritizations. In addition to informing funding and project implementation, prioritizations can help advise outreach efforts. Conservation practices are not self-adopting: Instead direct interaction with landowners where they are shown how participation fits into their existing operation is key (Kraft et al., 2003), as is growing social networks in high priority areas (Baumgart-Getz et al., 2012). Given the difficulties in increasing enrollment in conservation programs, spatial targeting may be followed by social targeting of landowners/farmers who would be more willing to participate. The suggestions provided here are ways to use targeting without fundamentally altering the incentive structures of current, voluntary conservation programs, but more flexibility in incentive structures has the potential to optimize adoption rates by farmers (Hao et al., 2023).

The goal of spatial targeting is to help move adoption of conservation practices beyond opportunistic enrollment to the lands where conservation is needed the most. In practice, however, many factors beyond nutrient reduction potential and environmental metrics are considered during prioritization work. Institutional readiness and the presence of a social network of willing conservation adopters is one such factor discussed earlier. Another factor is economics: For example, land prices and crop production are high in the corn producing areas of the Midwest (Upper Mississippi and Ohio basins), so taking land out of production to restore as wetlands is not likely, despite the alignment between surplus and potentially restorable wetlands in that region. A next step would be to incorporate economics or a scenario optimization (e.g., Hansen et al., 2021; Hao et al., 2023; Rabotyagov et al., 2014) into the prioritization framework. Nevertheless, having some sense of where and what kind of nutrient reduction is needed most – absent the economic and social constraints – is helpful to give decision-makers context as they tackle the “wicked” problem of excess nutrients from agriculture.

We recognize that the temporal scope of the data layers used in this analysis overlapped but did not perfectly align. Given our objective of describing nutrient reduction conservation needs at a broad, but coarse scale, we felt the data in combination would still lead to accurate overall spatial patterns, if not precision. At the time of this writing, more recent NNI data were not yet available, and we chose to use the mean of three years (2002, 2007, 2012) for surplus and efficiency metrics (versus only 2012) to better capture longer-term nutrient use trends and the need to mitigate legacy nutrients. Agricultural patterns are constantly shifting in response to economic forces: Between 2003 and 2016 (coinciding with the temporal range of data layers used in this analysis), agriculture in the US has shifted to larger, more specialized farms that planted more corn and soybean acreage and larger animal feeding operations that relied on imported feed (Conservation Effects Assessment Project, 2022). These and other changes in cropping and animal operation patterns have likely affected spatial patterns in surplus and efficiency, which could adjust which areas are identified as priorities for nutrient reduction and whether the conservation strategy would be better to focus on in-field or edge-of-field measures. Likewise, changing agricultural spatial patterns could impact landscape metrics such as nonbuffered agriculture, further adjusting recommended conservation strategies. Prioritizations using empirical data are probably most accurate, but due to the time lag until data become available, prioritizations are often based on older data, not current conditions. Put another way, more up-to-date information would be useful to provide more timely recommendations.

Despite the limitations in scope and data, we believe our approach for prioritizing nutrient reduction strategies in subwatersheds can be useful to conservation program managers, and we wanted to demonstrate it for a national example using readily available HUC8-level data. For managers working at the river basin or smaller scale, the accompanying dataset is structured to be easily filtered for the region of interest. We emphasize that nuances in interpreting these prioritizing metrics should be considered, often supplemented with local knowledge, and point out that the spatial distribution of the metrics could look rather differently with finer spatial scale data. Ultimately the work of targeted conservation will have to occur at those smaller spatial scales, and to that end, work is underway to produce a HUC12-level National Nutrient Inventory for 2018–2019. As finer scale data become available, the methods we developed here could be applied at smaller spatial scales to inform more local nutrient reduction efforts. (Note: The temporal scope of data layers would ideally be aligned when analyzing finer scale data.) Higher resolution data has shown to help increase nutrient reductions in the landscape (Singh et al., 2019) using tools like the Agricultural Conservation Planning Framework (ACPF; Tomer et al., 2013). Ideally, targeted approaches could even apply to field scales, although managers are often wary about focusing on individual farms within smaller watersheds despite evidence that US farmers could be open to recommendations from a targeted approach (Kalcic et al., 2014). Targeted outreach can also provide data critical to decision-making (Berkowitz et al., 2020), even if it does not immediately translate into conservation practice adoption. If we can spatially target nutrient reduction efforts on agricultural lands from national to field scales, we will be much more effective at decreasing nutrients reaching water bodies and much more likely to see definitive gains in water quality.

## 5. Conclusions

To help target conservation resources for reduction of agricultural nutrient release, we prioritized HUC8-level subwatersheds across CONUS using national nutrient inventory and agricultural landscape metrics. By utilizing nutrient surplus and nutrient use efficiency, we were able to not only identify where conservation efforts can focus, but also which types of conservation practices, singularly or in combination, might mitigate the excess nutrients. Our prioritization framework (Fig. 1) and spatial data analysis emphasize the substantial role that in- field conservation (i.e., reducing nutrient sources + preventing nutrients from leaving fields) may play in reducing nutrient inputs into the nation’s waters, as well as the many opportunities for edge-of-field conservation, like managing drainage effluent and buffering agricultural fields with natural vegetation, to complement in-field conservation (Fig. 6). We envision national, state, and watershed managers using the results of this national prioritization example and the accompanying dataset to inform and complement their own nutrient reduction prioritizations and help spatially target subwatersheds for conservation projects and enhanced outreach efforts.

## Supplementary Material

SI

## Figures and Tables

**Table 1:** Summary of data layers used in analysis. All layers had coverage for the conterminous US.

Metric/Data source/Publication	Spatial/temporal resolution	Underlying data	Description and highlights
**Agricultural nutrient surplus, Nitrogen use efficiency (NUE), Phosphorus use efficiency (PUE), Legacy P National Nutrient Inventory (NNI)** Sabo et al. (2021a)	HUC8 Mean of 2002, 2007, 2012	International Plant Nutrient Institute’s (IPNI) Nutrient Use Geographic Information System, Census of Agriculture (see Sabo et al., 2019; Sabo, Clark et al. 2021a,b for others)	Agricultural N surplus was calculated as the difference between farm-associated inputs (synthetic fertilizer, cultivated biological N fixation, atmospheric NO_x_ deposition onto farmland, livestock waste) and outputs (crop removal), and as such, represents both crop and livestock agriculture. Agricultural P surplus was similarly calculated except it excluded fixation and deposition. Legacy P are P surpluses summed from 1945 to 2001. Efficiency was calculated as the ratio between agricultural outputs and inputs. Both surplus and efficiency calculations are discussed in the Supplementary Materials T1.
**Nonbuffered agriculture EnviroAtlas** Christensen et al. (2013), Baker et al. (2006)	HUC12 2006–2010	National Land Cover Database (NLCD), Cropland Data Layer (CDL), National Hydrography Dataset NHDPlus HR, 30 m National Elevation Data	Nonbuffered agriculture was defined as having run-off flowpaths (following the topographic gradient) that would not intersect natural land cover before reaching a stream.
**Potentially restorable wetlands on agricultural land (PRW) EnviroAtlas** Horvath et al. (2017)	HUC12 2011–2014	NLCD, Soil Survey Geographic Database (SSURGO), 30 m National Elevation Data	This metric reflected agricultural land that may be suitable for wetland restoration. PRW naturally accumulate water and have some proportion of poorly drained soils. By considering both soils and landscape position, EnviroAtlas has more stringent criteria for their high PRW category than other studies (e.g., Evenson et al., 2021). Thus, we felt justified to include moderate and low PRW areas as well.
**Area, % Land EnviroAtlas** Pickard et al. (2015)	HUC12 2015	NHDPlus v2 Watershed Boundary Dataset Snapshot	This metric did not include subwatershed area outside the conterminous US.
**% Cropland, % Pasture EnviroAtlas** Pickard et al. (2015)	HUC12 2016	CDL	These metrics gave the percent of land managed as cropland or pasture. Pasture areas are planted for livestock grazing or the production of seed or hay crops. % cropland and % pasture were summed to get % agriculture.
**Tile drainage on agricultural land AgTile-US** Valayamkunnath et al. (2020)	30 m 2016–2019	NLCD, SSURGO, Shuttle Radar Topography Mission (SRTM) derived elevation, Census of Agriculture	Prediction map of tile drainage ground-truthed at 16,000 locations with 86% accuracy nationwide and up to 94% accuracy in the Midwest.

## Data Availability

Data is publicly available at ScienceHub doi:10.23719/1529848
