# Supplementary material for: Our national nutrient reduction needs: Applying a conservation prioritization framework to US agricultural lands
Source: J Environ Manage. Author manuscript; Available in PMC 2025 Feb 1. (PMC10851882; doi:10.1016/j.jenvman.2023.119758)
Supplement: SI [file NIHMS1952785-supplement-SI.docx]

**SUPPLEMENTARY MATERIALS**


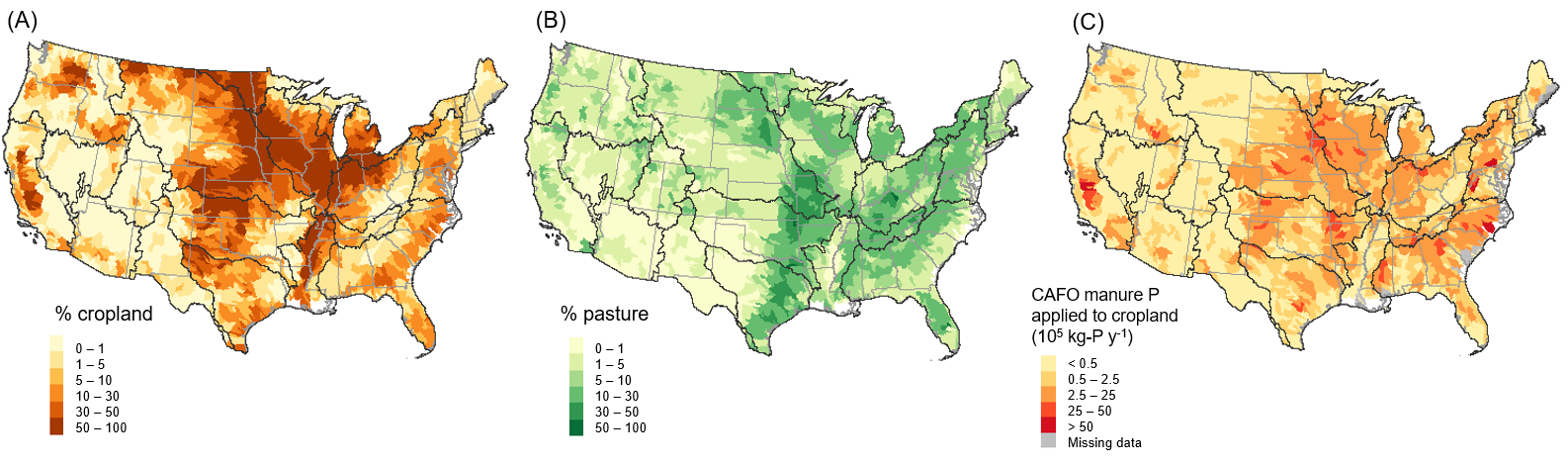


Figure S1 – Spatial distribution of the different types of agriculture across the conterminous US (CONUS). Crop (A) and grazed livestock (B) are presented as percentage of HUC8 subwatershed area based on land cover. Manure applied to crop fields is used as a proxy for confined animal feeding operations (CAFO) density (C). States are outlined in light grey and HUC2 river basins are outlined in black on this and subsequent maps.

Table S1 – Agricultural area within HUC2 river basins. Basins are ordered from the highest number of high N and/or P surplus subwatersheds to the lowest.

| **HUC2 river basin** | **Agricultural land (km^2^)** | **Land area (km^2^)** |
| --- | --- | --- |
| Missouri | 467000 | 1303000 |
| South Atlantic-Gulf | 126000 | 660000 |
| Arkansas-White-Red | 198000 | 634000 |
| Upper Mississippi | 295000 | 478000 |
| Texas-Gulf | 125000 | 459000 |
| Ohio | 158000 | 417000 |
| Pacific Northwest | 80000 | 703000 |
| California | 46000 | 410000 |
| Great Lakes | 103000 | 315000 |
| Pacific Northwest | 80000 | 703000 |
| Lower Mississippi | 91000 | 230000 |
| Mid-Atlantic | 54000 | 237000 |
| Souris-Red-Rainy | 87000 | 146000 |
| Tennessee | 25000 | 103000 |
| Lower Colorado | 9000 | 362000 |
| New England | 9000 | 149000 |
| Rio Grande | 8000 | 343000 |
| Great Basin | 12000 | 360000 |
| Upper Colorado | 9000 | 292000 |

**T1 Surplus and NUE**

The agricultural nutrient surplus (henceforth “surplus”, *Surplus*) equation used by the National Nutrient Inventory (NNI) is derived (Equation S3). Technically, agricultural inputs are synthetic fertilizer (*Fert*), cultivated biological N fixation (*Bio*), atmospheric NO_X_ deposition onto farmland (*Atm*), and livestock feed demand (*LFeed*). Agricultural outputs are crop removal (*Crop*) and livestock production (*LProd*). Together,

|  | $Surplus=Fert+Bio+Atm+LFeed-LProd-Crop$ | *(Eq. S1)* |
| --- | --- | --- |

Since manure is an input and output and represents an internal cycling of nutrients, it was not included in the surplus equation. The NNI uses a simple (vs dynamic) livestock model where

|  | $LFeed=LProd+LWaste$ | *(Eq. S2)* |
| --- | --- | --- |

Substituting livestock waste (*LWaste*) for $LFeed-LProd$ yields the derived surplus equation:

|  | $Surplus=Fert+Bio+Atm+LWaste-Crop$ | *(Eq. S3)* |
| --- | --- | --- |

Nutrient use efficiency (henceforth “efficiency”) was calculated by the NNI as

|  | $\frac{Crop}{Fert+Bio+Atm+LWaste}$ | *(Eq. S4)* |
| --- | --- | --- |

based on the derived surplus equation S3. Technically, efficiency is outputs divided by inputs or

|  | $\frac{Crop+LProd}{Fert+Bio+Atm+LFeed}$ | *(Eq. S5)* |
| --- | --- | --- |

The difference between these two ways to calculate efficiency is not trivial because it disproportionately affects subwatersheds with lower efficiency (Figure S2).


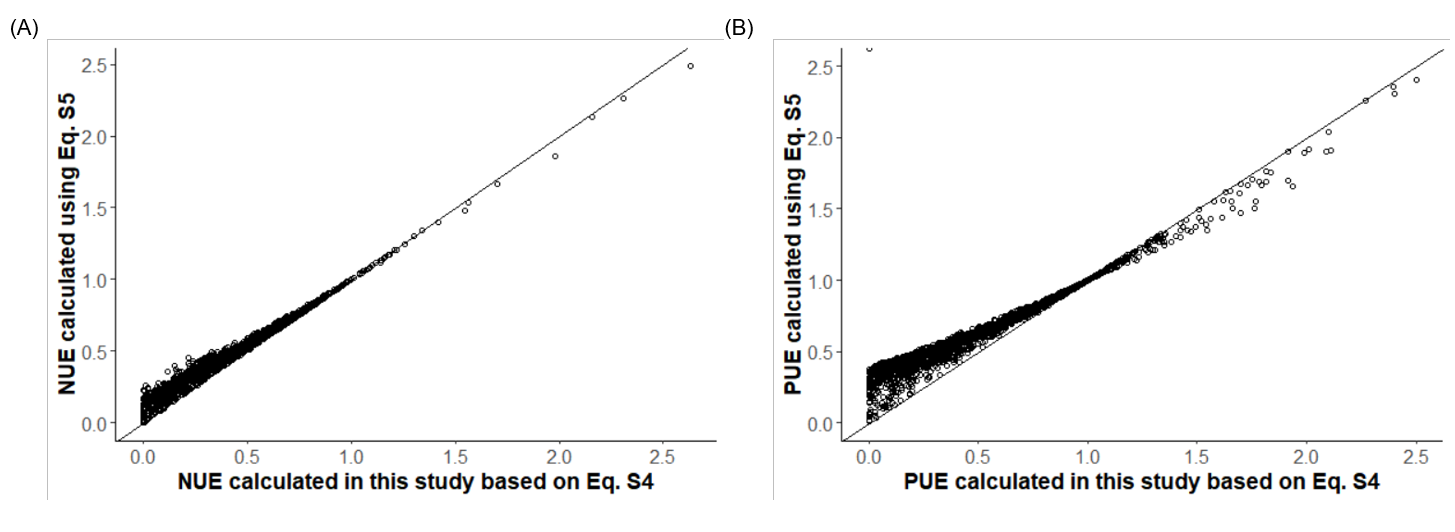


Figure S2 – Comparison of nitrogen and phosphorus use efficiency (NUE and PUE, respectively) calculated using the full (y-axis) and derived (x-axis) surplus equations.

**T2 Breakpoints for color classes used in figures**

(Note: Literature values used to determine breakpoints are also landscape-level areal rates, except where otherwise noted.)

The breakpoints selected for N surplus were 1, 7, 15, and 55 kg-N ha^-1^ y^-1^. The value of 1 kg-N ha^-1^ y^-1^ reflects the lower boundary for N critical loads for lower-order organisms such as diatoms, algae, lichen, and bryophytes (Pardo et al., 2011), above which we start to see ecological effects of increased N, as well as mean atmospheric N deposition in the averaged NNI data. 7 kg-N ha^-1^ y^-1^ reflects the highest atmospheric N deposition, as well as the median N surplus (7.4 kg-N ha^-1^ y^-1^). 15 kg-N ha^-1^ y^-1^ was the 70^th^ percentile for N surplus in the averaged NNI data (Sabo et al., 2021a). At around 55 kg-N ha^-1^ y^-1^, the probability of groundwater drinking water violations in the USA begins to increase more rapidly with an inflection point at 75 kg-N ha^-1^ y^-1^ (Pennino et al., 2020). We focused our analyses on subwatersheds in the highest three N surplus classes, considering them “high” N surplus subwatersheds.

The breakpoints selected for P surplus were 0.25, 1, 2, and 8 kg-P ha^-1^ y^-1^. The lowest breakpoint of 0.25 kg-P ha^-1^ y^-1^ reflects the highest atmospheric P deposition in the NNI data (Sabo et al., 2021b) as well as the 25^th^ percentile of the averaged NNI P surplus data. 1 kg-P ha^-1^ y^-1^ reflects the median P surplus in the averaged NNI data, as well as high field-scale atmospheric P deposition for agriculture in the literature (Redfield, 2002). 2 and 8 kg-P ha^-1^ y^-1^ reflect the 70^th^ and 95^th^ percentile for P surplus in the averaged NNI data. Again, we focused our analyses on subwatersheds in the highest three P surplus classes, considering them “high” P surplus subwatersheds.

Multiple studies seem to agree that the upper bound for both nitrogen and phosphorus use efficiencies (NUE and PUE, respectively) for sustainable agricultural systems is 0.90 – 0.95 (McCrackin et al., 2018; Van Meter et al., 2021; Zhang et al., 2015), assuming no soil mining of nutrients. Zhang et al. (2015) suggest the USA should have target NUE set slightly above other regions of the world at 0.75; however, we took a more conservative approach by using an efficiency of 0.70 which reflects a) the global nitrogen use efficiency (NUE) average promoted by Zhang et al. (2015) to meet environmental stewardship goals without compromising food security and b) the ~75^th^ percentiles for both NUE and PUE in the NNI data. To be clear, it is possible for farmers to achieve even greater efficiency – up to 80 or 90% in certain cropping systems – but this effort looks to identify subwatersheds where efficiency is particularly low. The breakpoints of 0.5 and 0.3 efficiency are slightly above the 50^th^ and 25^th^ percentiles for NUE (0.46 and 0.26, respectively) in CONUS. The 50^th^ and 25^th^ percentiles for PUE are lower at 0.41 and 0.19.

Estimates of tile drainage vary across CONUS, with highest average values in the Midwest (e.g., 37% of cropland in the Midwest (King et al., 2015), 25-35% of cropland in Iowa (Schilling and Helmers, 2008)). The breakpoints for tile drainage start at 30% (roughly the 95^th^ percentile for CONUS), or what we consider “highly” tiled areas in our national study.

The breakpoints of 2% and 15% of the watershed with nonbuffered agriculture were chosen to capture the 40^th^ and 80^th^ percentiles for CONUS. We chose 2% as our threshold to differentiate between well-buffered agriculture (<2%) and agriculture that could use buffering (≥2%).

The breakpoints of 3%, 10%, and 30% of agricultural land that are PRW reflect roughly the 25^th^, 50^th^, and 75^th^ percentiles of subwatersheds in CONUS, including 362 subwatersheds that had no PRW. We chose 10% as our threshold to differentiate between where wetland restoration would realistic be part of conservation strategies (≥10%) and where it would not (<10%).


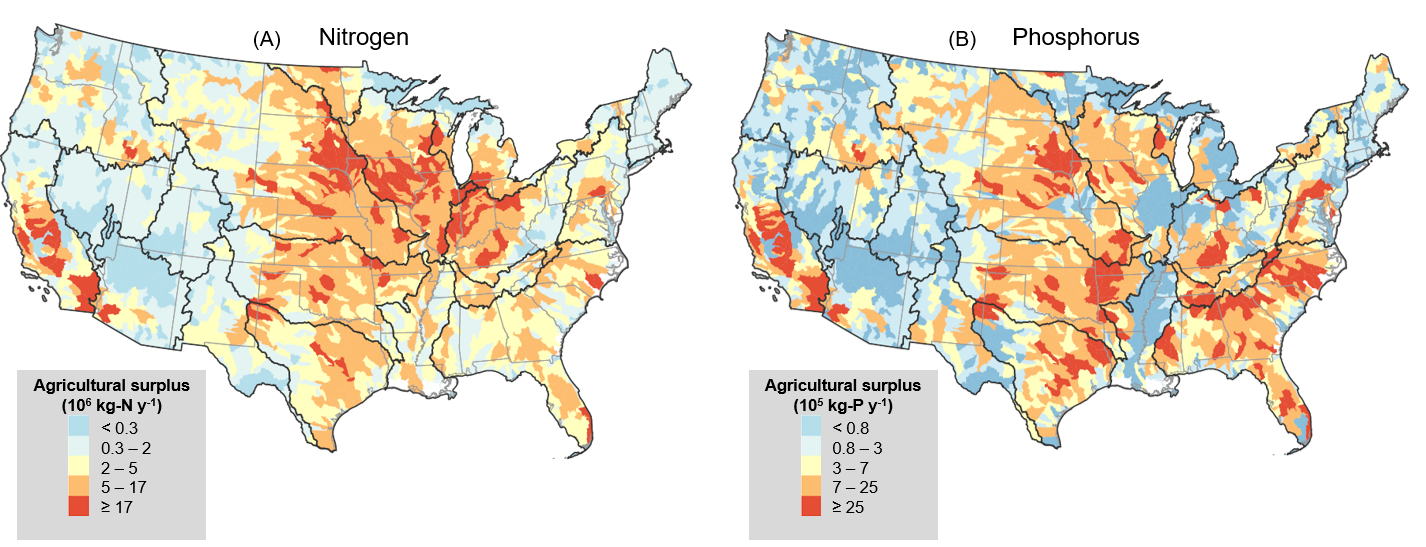


Figure S3 – Agricultural nutrient surplus as total HUC8 subwatershed rates for nitrogen (A) and phosphorus (B) across CONUS. Surplus classes are based on the areal normalized thresholds described earlier multiplied by the median HUC8 subwatershed area (3160 km^2^) and rounded to the nearest million kg-N y^-1^ or nearest 10^5^ kg-P y^-1^.

**T3 Nutrient use efficiency thresholds**

In regions of interest with efficiencies ≥70% and high surpluses, the efficiency threshold could be raised, even as conservation strategies focus heavily on preventing nutrients from leaving fields and trapping/treating nutrients beyond the field. These high efficiency subwatersheds are primarily located in the Midwest due to well-refined nutrient management of its major crops (corn/soybean rotation; see Swaney et al., 2018). However despite the fine-tuning of fertilizer use efficiency, crop agriculture intensity in the Midwest is so great that surpluses remain exceptionally high, especially for N (Figure 3A), explaining the lack of strong negative correlation between NUE and N surplus nationally. These exceptionally high surplus areas are perhaps most in need of combined in-field and edge-of-field conservation to ameliorate the high amounts of excess nutrients, and conservation can include continued efforts to reduce nutrient inputs and further raise efficiencies towards 0.90, the upper bound for NUE and PUE for sustainable agricultural systems (McCrackin et al., 2018; Van Meter et al., 2021; Zhang et al., 2015). Upward adjustment of efficiency goals, however, should be limited due to farmer hesitancy about potential reductions to crop yields with even lower fertilizer applications.

For other agricultural areas and types, efficiency goals may likely need downward adjustment. Where agriculture has a large livestock component, not all livestock waste is recovered as manure fertilizer for crops, thus leading to lower efficiencies. The fraction of livestock waste recovered as manure fertilizer is ~75% for CONUS for N (Sabo et al., 2019) and ~65% for P (Sabo et al., 2021b), but can vary widely depending on livestock type and whether the livestock is pastured or confined. For poultry and hogs that spend most of their time in confined animal feeding operations (CAFOs), almost all waste can be recovered, minus manure handling losses due to ammonia volatilization and leaching during storage, which is minimal for P. However, pastured livestock such as cattle and horses have much lower recovery fractions (Conant et al., 2013). For all these reasons, NUE and PUE both tend to be lower in areas where agriculture is dominated by livestock (vs cropland) both in the US (Swaney and Howarth, 2019; Swaney et al., 2018) and abroad (McCrackin et al., 2018). As a consequence, an efficiency goal of 0.7 may be unrealistic for some pastured livestock systems (though it is unlikely these subwatersheds are in the top quartile for surplus), and as a result of lower efficiency goals, more subwatersheds might have met efficiency thresholds than we have suggested, and hence the areas where strategies would focus more on edge-of-field conservation would have expanded. There are some other nuances to the calculation of efficiency that disproportionately affect livestock-heavy systems: They are discussed in the Supplementary Materials T1. The effects of recovery fractions and calculation method, however, should not downplay the many inefficiencies in livestock agriculture that lead to nutrient run-off into water bodies. Unfortunately, it is common that livestock waste is applied as manure fertilizer, but that recycling effort is not always accompanied by decreases in synthetic fertilizer application (Conservation Effects Assessment Project, 2022). Increasing the efficiency of the combined agricultural system (i.e. crops that generate feed and the livestock operations themselves), shrinking the size of manuresheds (Spiegal et al., 2020), and reducing synthetic fertilizer applied to fields in parallel with livestock waste being recovered and used as manure fertilizer are all in-field conservation measures that could greatly reduce excess nutrients that could be delivered to water bodies from livestock-heavy systems (Flynn et al., 2023).


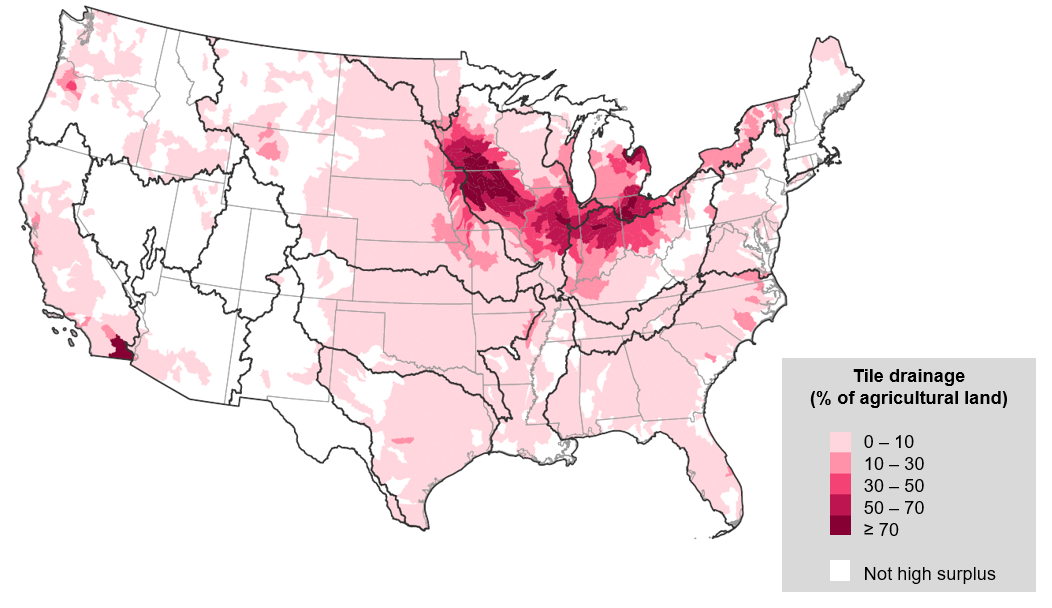


Figure S4 – Tile drainage for high surplus subwatersheds across CONUS.


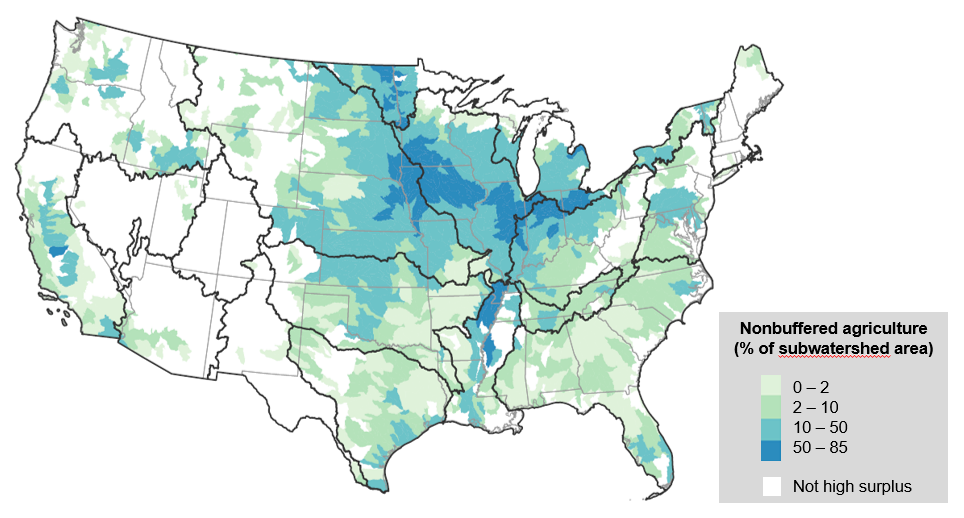


Figure S5 – Nonbuffered agriculture for high surplus subwatersheds across CONUS.


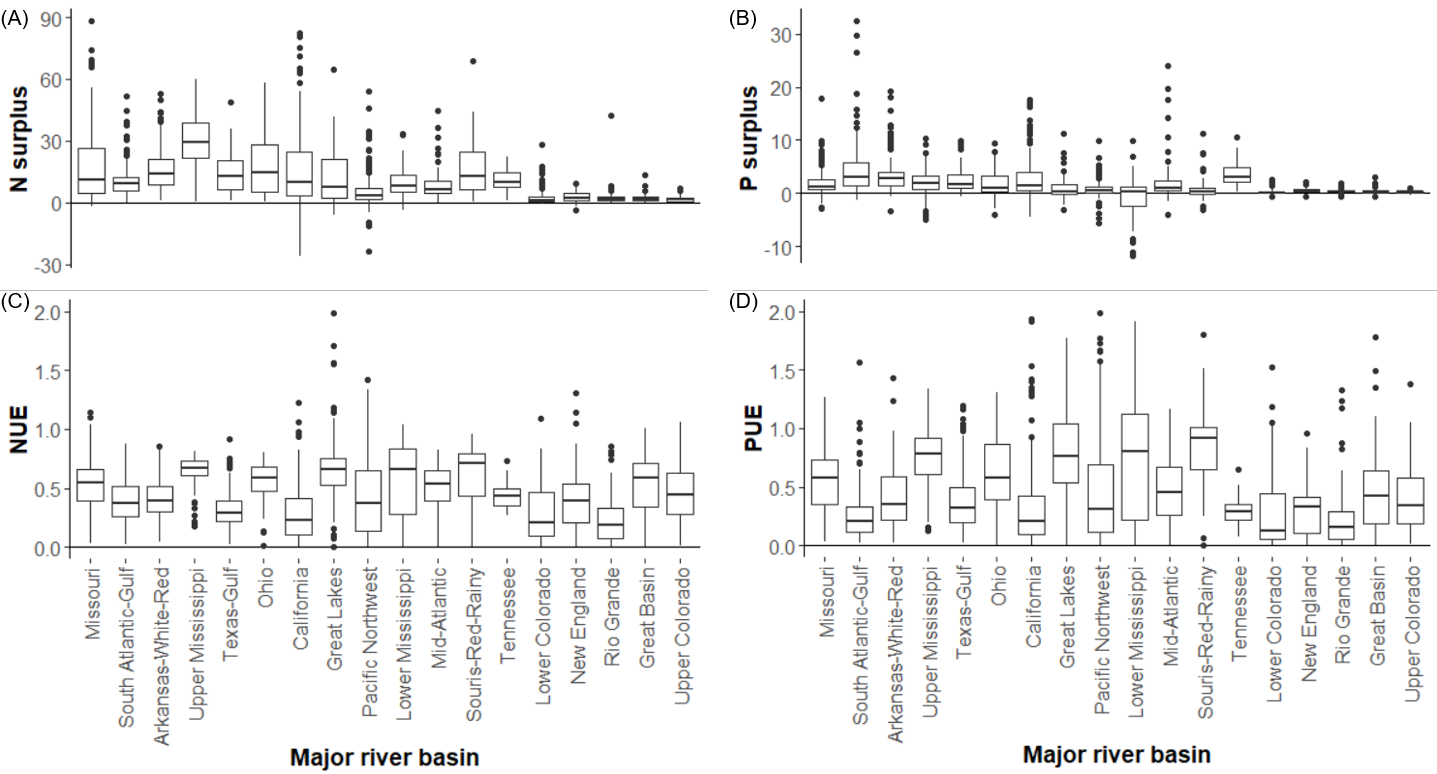


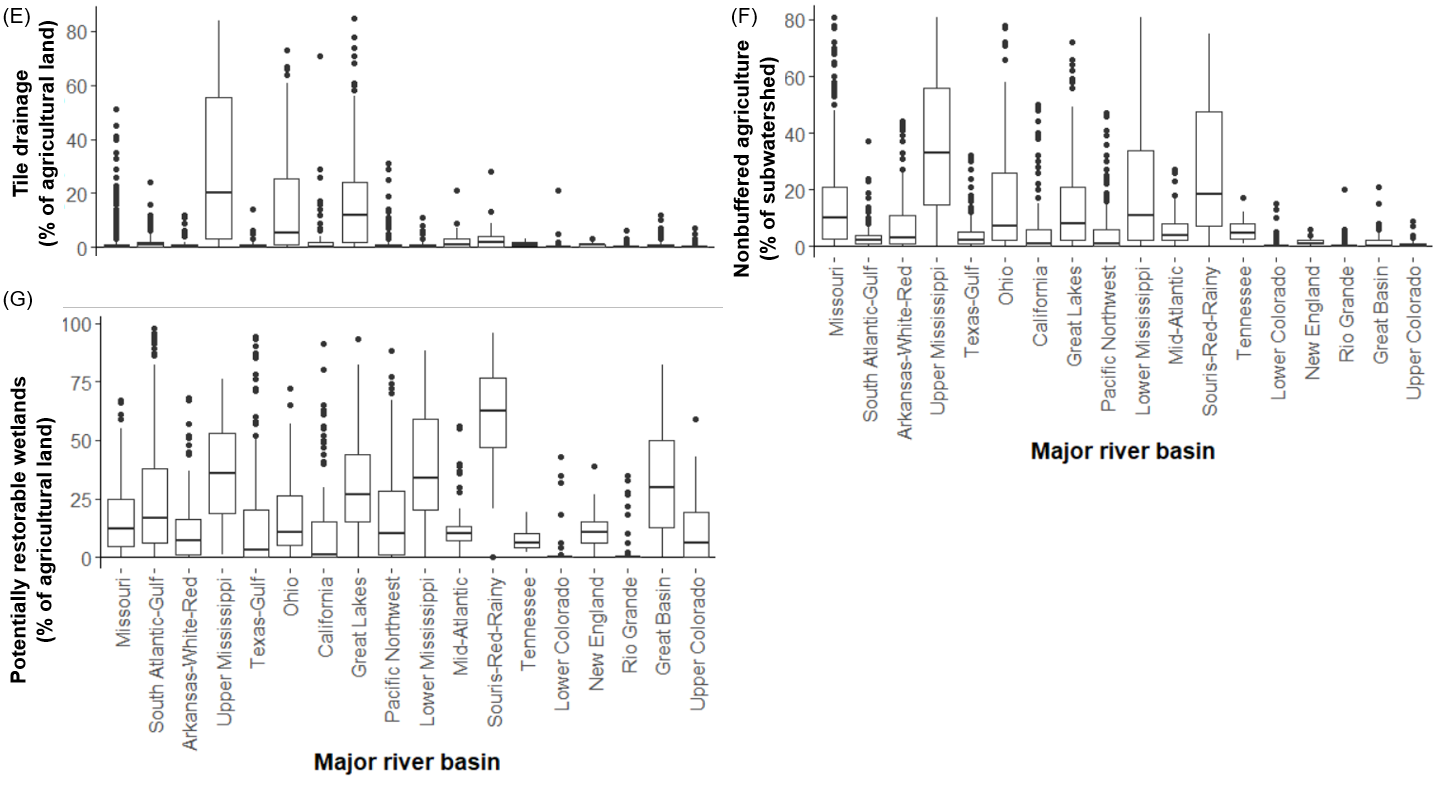


Figure S6 – Distribution of nutrient use and agricultural landscape metrics within and across HUC2 river basins. NUE and PUE stand for nitrogen and phosphorus use efficiency, respectively.

Table S2 – Spearman rank correlation matrix for nutrient use and agricultural land metrics considered in this study. NUE and PUE stand for nitrogen and phosphorus use efficiency, respectively, and PRW refers to potentially restorable wetlands.

|  | Agricultural area | Cropland area | Pasture area | N surplus | P surplus | NUE | PUE | Tile-drained area | Nonbuffered agriculture area | PRW area |
| --- | --- | --- | --- | --- | --- | --- | --- | --- | --- | --- |
| Agricultural area | 1 | 0.93 | 0.68 | 0.68 | 0.30 | 0.59 | 0.61 | 0.73 | 0.91 | 0.80 |
| Cropland area | 0.93 | 1 | 0.45 | 0.66 | 0.22 | 0.62 | 0.66 | 0.71 | 0.92 | 0.74 |
| Pasture area | 0.68 | 0.45 | 1 | 0.41 | 0.36 | 0.36 | 0.30 | 0.56 | 0.55 | 0.60 |
| N surplus | 0.68 | 0.66 | 0.41 | 1 | 0.65 | 0.10 | 0.20 | 0.62 | 0.62 | 0.55 |
| P surplus | 0.30 | 0.22 | 0.36 | 0.65 | 1 | -0.29 | -0.36 | 0.25 | 0.15 | 0.17 |
| NUE | 0.59 | 0.62 | 0.36 | 0.10 | -0.29 | 1 | 0.91 | 0.48 | 0.67 | 0.58 |
| PUE | 0.61 | 0.66 | 0.31 | 0.20 | -0.36 | 0.91 | 1 | 0.50 | 0.71 | 0.58 |
| Tile-drained area | 0.73 | 0.71 | 0.56 | 0.62 | 0.25 | 0.48 | 0.50 | 1 | 0.72 | 0.69 |
| Nonbuffered agriculture area | 0.91 | 0.92 | 0.55 | 0.62 | 0.15 | 0.67 | 0.71 | 0.72 | 1 | 0.79 |
| PRW area | 0.80 | 0.74 | 0.60 | 0.55 | 0.17 | 0.58 | 0.58 | 0.69 | 0.79 | 1 |

**T4 Wetland restoration**

Where edge-of-field conservation is a focus of nutrient reduction strategies, restoring wetlands to buffer agricultural run-off may be one of the more effective options. Unlike other non-wetland buffers, the anaerobic conditions in wetland soils allow biogeochemical processes (e.g. denitrification) to transform – and thereby remove – nutrients. The effectiveness of wetlands in removing nutrients is well-documented at the individual wetland scale (Crumpton et al., 2020; Mitsch et al., 2005), and modeling work suggests that wetlands may be effective at larger watershed (Hansen et al., 2018; Pinardi et al., 2020), regional (Evenson et al., 2021; Singh et al., 2019) and national scales (Cheng et al., 2020). Restoring wetlands on former agricultural lands does take land out of agricultural production (i.e. opportunity cost, although the land is often of marginal productivity) and may require up-front investment to return the hydrology to its natural state (i.e. restoration cost, although not nearly as high as for created wetlands). However, because of the high potential benefits of wetlands relative to other conservation practices (excluding nutrient management), restoration becomes quite cost-effective compared to other conservation practices like cover crops and two-stage ditches (Roley et al., 2016), despite its higher initial cost.

Since wetlands simultaneously but differentially affect both N and P, evaluating both nutrients together when considering wetland restoration can prevent pollution swapping (the unintentional increase in other pollutants when trying to mitigate a target pollutant). High N surplus and high P surplus areas are often co-located (Sabo et al 2021), and wetlands have been shown to simultaneously reduce both (e.g. Woltemade, 2000). However, while the anaerobic environment of waterlogged soils promotes denitrification, it also promotes iron reduction which may lead to the release of P from soils into the water depending on water column P concentrations (Ardón et al., 2010), causing wetlands to be a P source immediately upon flooding and over longer time scales (Sharpley et al., 2013). With only plant uptake to remove the newly released P, and given the large stores of legacy nutrients in agricultural soils (Van Staden et al., 2021), there is a concern that restored wetlands can release P for years if not decades, supported by evidence that P source behavior occurs more often in constructed/restored wetlands than natural ones (Land et al., 2016). Many of the opportune areas for wetland restoration based on other metrics also have high legacy P in agricultural field soils (Figure S7). The factors that influence legacy P release by restored wetlands, their spatial distribution, and how it shapes restoration targeting is a topic for future work. Given uncertain effects on P loads of wetland restoration in places likely to release legacy P, restoring wetlands is currently not recommended to be considered as part of conservation strategies unless there are high N surpluses. Otherwise, high legacy P may not necessarily preclude wetland restoration if erosion of agricultural soils is less likely or if management practices can minimize erosion. Strategies in these high N surplus subwatersheds where restored wetlands are useful to buffer agricultural run-off could include soil conservation efforts and stacking erosion prevention practices (e.g. grassed waterways, buffer strips) with wetland restoration to reduce the likelihood soil-bound P will be released under the anaerobic conditions in the wetlands. That said, Dodd and Sharpley (2015) cautioned that conservation practices themselves may become a source of legacy P over time, and Yuan et al. (2022) pointed out a research gap regarding the effectiveness of conservation practices in reducing dissolved P. Obviously, the potential release of legacy P must be a consideration when restoring wetlands (Nair and Harris, 2014) and is another reminder of the primacy of improving in-field efficiency before relying on edge-of-field conservation.


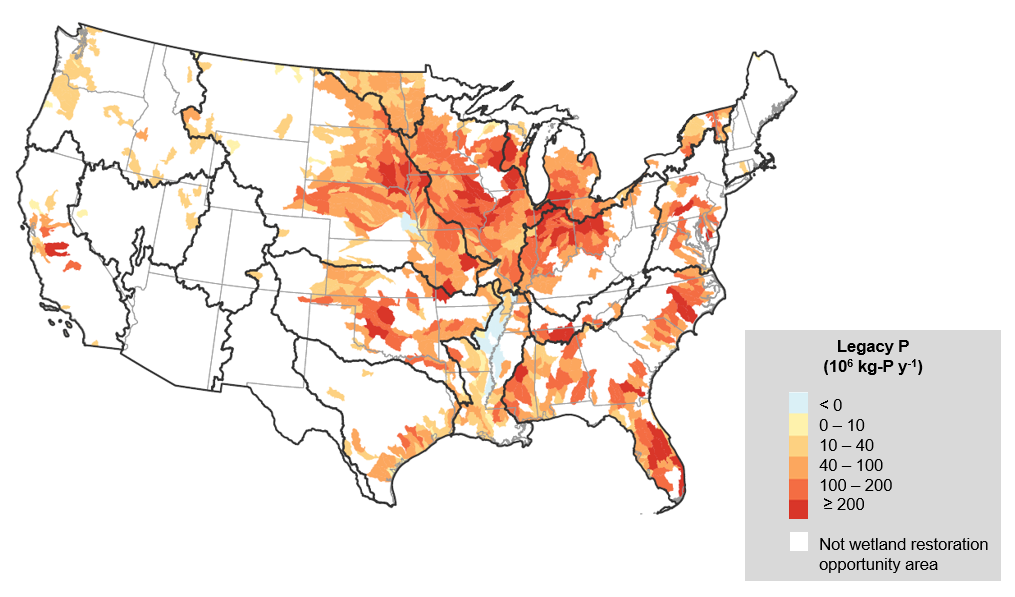


Figure S7 – Agricultural legacy P (sum of agricultural P surpluses for years 1945 – 2001) for subwatersheds with high surplus as well as potentially restorable wetlands ≥10% of agricultural land. Legacy P data notably did not incorporate losses due to erosion, leaching, or surface water loading. We used the high end of the legacy P estimates from Sabo et al. (2021b) primarily for two reasons. The first is that it is conservative and captures the likely maximum amount of phosphorus present within a subbasin. Second, the crop removal ratio to help derive the legacy estimates relied on crop removal and nutrient input values from 1987, 1992, and 1997. However, fertilizer P inputs largely peaked in the early to mid-1980s, and there has been a steady increase in phosphorus use efficiency since then. Thus, median legacy estimates are likely biased low since phosphorus use efficiency was lower pre-1987. For more details regarding the legacy P assumptions and calculations, see Sabo et al. (2021b).

**T5 Other considerations when spatially targeting for nutrient reduction**

When managing for downstream impacts, subwatershed position in the river network may be an important consideration for prioritization efforts. By modeling the supply and transport of N and P, Alexander et al. (2008) showed a dendritic spatial pattern of nutrient delivery to the Gulf of Mexico from the Mississippi river basins, with higher nutrient yields originating from lands closer to the main stem of the Mississippi River and her largest tributaries. They attribute the dendritic pattern to the combined effects of two longitudinal trends within the river channel: 1) less nutrient removal in deeper waters and 2) higher water velocities (i.e. shorter residence times) as stream order increases. Based on these findings, prioritizing conservation efforts near large rivers or smaller streams that flow quickly to large rivers may achieve the greatest reductions in lotic nutrient loads. Even so, Alexander et al. (2008) also reported larger fractions of N originating from smaller streams than previous studies – a caution for managers not to discount headwater basins for conservation. Our study’s national scope did not focus on a specific receiving water like the Great Lakes or Gulf of Mexico, but with the accompanying dataset, managers can sort by HUC2 river basin and then prioritize subwatersheds considering surplus, efficiency, drainage, existing buffers, PRW, and additionally, river network position.

**References for Supplementary Materials**

Alexander, R.B., Smith, R.A., Schwarz, G.E., Boyer, E.W., Nolan, J.V., Brakebill, J.W., 2008. Differences in phosphorus and nitrogen delivery to the Gulf of Mexico from the Mississippi River Basin. Environmental Science and Technology 42, 822-830.

Ardón, M., Montanari, S., Morse, J.L., Doyle, M.W., Bernhardt, E.S., 2010. Phosphorus export from a restored wetland ecosystem in response to natural and experimental hydrologic fluctuations. Journal of Geophysical Research 115.

Cheng, F.Y., Van Meter, K.J., Byrnes, D.K., Basu, N.B., 2020. Maximizing US nitrate removal through wetland protection and restoration. Nature 588, 625-630.

Conant, R.T., Berdanier, A.B., Grace, P.R., 2013. Patterns and trends in nitrogen use and nitrogen recovery efficiency in world agriculture. Global Biogeochemical Cycles 27, 558-566.

Conservation Effects Assessment Project, 2022. Conservation practices on cultivated cropland: A comparison of CEAP I and CEAP II survey data and modeling. CEAP National Report, 165.

Crumpton, W.G., Stenback, G.A., Fisher, S.W., Stenback, J.Z., Green, D.I.S., 2020. Water quality performance of wetlands receiving nonpoint-source nitrogen loads: Nitrate and total nitrogen removal efficiency and controlling factors. J Environ Qual 49, 735-744.

Dodd, R.J., Sharpley, A.N., 2015. Conservation practice effectiveness and adoption: unintended consequences and implications for sustainable phosphorus management. Nutrient Cycling in Agroecosystems 104, 373-392.

Evenson, G.R., Golden, H.E., Christensen, J.R., Lane, C.R., Rajib, A., D'Amico, E., Mahoney, D.T., White, E., Wu, Q., 2021. Wetland restoration yields dynamic nitrate responses across the Upper Mississippi river basin. Environ Res Commun 3, 1-10.

Flynn, K.C., Spiegal, S., Kleinman, P.J.A., Meinen, R.J., Smith, D.R., 2023. Manureshed management to overcome longstanding nutrient imbalances in US agriculture. Resources, Conservation and Recycling 188.

Hansen, A.T., Dolph, C.L., Foufoula-Georgiou, E., Finlay, J.C., 2018. Contribution of wetlands to nitrate removal at the watershed scale. Nature Geoscience 11, 127-132.

King, K.W., Williams, M.R., Fausey, N.R., 2015. Contributions of systematic tile drainage to watershed-scale phosphorus transport. J Environ Qual 44, 486-494.

Land, M., Granéli, W., Grimvall, A., Hoffmann, C.C., Mitsch, W.J., Tonderski, K.S., Verhoeven, J.T.A., 2016. How effective are created or restored freshwater wetlands for nitrogen and phosphorus removal? A systematic review. Environmental Evidence 5.

McCrackin, M.L., Gustafsson, B.G., Hong, B., Howarth, R.W., Humborg, C., Savchuk, O.P., Svanbäck, A., Swaney, D.P., 2018. Opportunities to reduce nutrient inputs to the Baltic Sea by improving manure use efficiency in agriculture. Regional Environmental Change 18, 1843-1854.

Mitsch, W.J., Day, J.W., Zhang, L., Lane, R.R., 2005. Nitrate-nitrogen retention in wetlands in the Mississippi River Basin. Ecological Engineering 24, 267-278.

Nair, V.D., Harris, W.G., 2014. Soil phosphorus storage capacity for environmental risk assessment. Advances in Agriculture 2014, 1-9.

Pardo, L.H., Fenn, M.E., Goodale, C.L., Geiser, L., Driscoll, C.T., Allen, E.B., Baron, J.S., Bobbink, R., Bowman, W.D., Clark, C.M., Emmett, B., Gilliam, F.S., Greaver, T.L., Hall, S.J., Lilleskov, E.A., Liu, L., Lynch, J.A., Nadelhoffer, K.J., Perakis, S.S., Robin-Abbott, M.J., Stoddard, J.L., Weathers, K.C., Dennis, R.L., 2011. Effects of nitrogen deposition and empirical nitrogen critical loads for ecoregions of the United States. Ecological Applications 21, 3049-3082.

Pennino, M.J., Leibowitz, S.G., Compton, J.E., Hill, R.A., Sabo, R.D., 2020. Patterns and predictions of drinking water nitrate violations across the conterminous United States. Sci Total Environ 722, 137661.

Pinardi, M., Soana, E., Bresciani, M., Villa, P., Bartoli, M., 2020. Upscaling nitrogen removal processes in fluvial wetlands and irrigation canals in a patchy agricultural watershed. Wetlands Ecology and Management 28, 297-313.

Redfield, G.W., 2002. Atmospheric deposition of phosphorus to the everglades: concepts, constraints, and published deposition rates for ecosystem management. ScientificWorldJournal 2, 1843-1873.

Roley, S.S., Tank, J.L., Tyndall, J.C., Witter, J.D., 2016. How cost-effective are cover crops, wetlands, and two-stage ditches for nitrogen removal in the Mississippi River Basin? Water Resources and Economics 15, 43-56.

Sabo, R.D., Clark, C.M., Bash, J., Sobota, D., Cooter, E., Dobrowolski, J.P., Houlton, B.Z., Rea, A., Schwede, D., Morford, S.L., Compton, J.E., 2019. Decadal shift in nitrogen inputs and fluxes across the contiguous United States: 2002–2012. Journal of Geophysical Research: Biogeosciences 124, 3104-3124.

Sabo, R.D., Clark, C.M., Compton, J.E., 2021a. Considerations when using nutrient inventories to prioritize water quality improvement efforts across the US. Environ Res Commun 3.

Sabo, R.D., Clark, C.M., Gibbs, D.A., Metson, G.S., Todd, M.J., LeDuc, S.D., Greiner, D., Fry, M.M., Polinsky, R., Yang, Q., Tian, H., Compton, J.E., 2021b. Phosphorus inventory for the conterminous United States (2002–2012). Journal of Geophysical Research: Biogeosciences 126.

Schilling, K.E., Helmers, M., 2008. Effects of subsurface drainage tiles on streamflow in Iowa agricultural watersheds: Exploratory hydrograph analysis. Hydrological Processes 22, 4497-4506.

Sharpley, A., Jarvie, H.P., Buda, A., May, L., Spears, B., Kleinman, P., 2013. Phosphorus legacy: overcoming the effects of past management practices to mitigate future water quality impairment. J Environ Qual 42, 1308-1326.

Singh, N.K., Gourevitch, J.D., Wemple, B.C., Watson, K.B., Rizzo, D.M., Polasky, S., Ricketts, T.H., 2019. Optimizing wetland restoration to improve water quality at a regional scale. Environmental Research Letters 14.

Spiegal, S., Kleinman, P.J.A., Endale, D.M., Bryant, R.B., Dell, C., Goslee, S., Meinen, R.J., Flynn, K.C., Baker, J.M., Browning, D.M., McCarty, G., Bittman, S., Carter, J., Cavigelli, M., Duncan, E., Gowda, P., Li, X., Ponce-Campos, G.E., Cibin, R., Silveira, M.L., Smith, D.R., Arthur, D.K., Yang, Q., 2020. Manuresheds: Advancing nutrient recycling in US agriculture. Agricultural Systems 182.

Swaney, D.P., Howarth, R.W., 2019. Phosphorus use efficiency and crop production: Patterns of regional variation in the United States, 1987-2012. Sci Total Environ 685, 174-188.

Swaney, D.P., Howarth, R.W., Hong, B., 2018. Nitrogen use efficiency and crop production: Patterns of regional variation in the United States, 1987-2012. Sci Total Environ 635, 498-511.

Van Meter, K.J., McLeod, M.M., Liu, J., Tenkouano, G.T., Hall, R.I., Van Cappellen, P., Basu, N.B., 2021. Beyond the mass balance: Watershed phosphorus legacies and the evolution of the current water quality policy challenge. Water Resources Research 57.

Van Staden, T.L., Van Meter, K.J., Basu, N.B., Parsons, C.T., Akbarzadeh, Z., Van Cappellen, P., 2021. Agricultural phosphorus surplus trajectories for Ontario, Canada (1961-2016), and erosional export risk. Sci Total Environ, 151717.

Woltemade, C.J., 2000. Ability of restored wetlands to reduce nitrogen and phosphorus concentrations in agricultural drainage water. Journal of Soil and Water Conservation.

Yuan, Y., Book, R.S., Mankin, K.R., Koropeckyj-Cox, L., Christianson, L., Messer, T., Christianson, R., 2022. An overview of the effectiveness of agricultural conservation practices for water quality improvement. J ASABE 65, 419-426.

Zhang, X., Davidson, E.A., Mauzerall, D.L., Searchinger, T.D., Dumas, P., Shen, Y., 2015. Managing nitrogen for sustainable development. Nature 528, 51-59.
